# Genome-Wide Chromatin Remodeling Identified at GC-Rich Long Nucleosome-Free Regions

**DOI:** 10.1371/journal.pone.0047924

**Published:** 2012-11-05

**Authors:** Karin Schwarzbauer, Ulrich Bodenhofer, Sepp Hochreiter

**Affiliations:** Institute of Bioinformatics, Johannes Kepler University, Linz, Austria; Roswell Park Cancer Institute, United States of America

## Abstract

To gain deeper insights into principles of cell biology, it is essential to understand how cells reorganize their genomes by chromatin remodeling. We analyzed chromatin remodeling on next generation sequencing data from resting and activated T cells to determine a whole-genome chromatin remodeling landscape. We consider chromatin remodeling in terms of nucleosome repositioning which can be observed most robustly in long nucleosome-free regions (LNFRs) that are occupied by nucleosomes in another cell state. We found that LNFR sequences are either AT-rich or GC-rich, where nucleosome repositioning was observed much more prominently in GC-rich LNFRs — a considerable proportion of them outside promoter regions. Using support vector machines with string kernels, we identified a GC-rich DNA sequence pattern indicating loci of nucleosome repositioning in resting T cells. This pattern appears to be also typical for CpG islands. We found out that nucleosome repositioning in GC-rich LNFRs is indeed associated with CpG islands and with binding sites of the CpG-island-binding ZF-CXXC proteins KDM2A and CFP1. That this association occurs prominently inside and also prominently outside of promoter regions hints at a mechanism governing nucleosome repositioning that acts on a whole-genome scale.

## Introduction

A major goal of biological research is to understand the dynamics of genome organization by chromatin remodeling which controls the access of proteins to DNA and thereby transcription [1]–[9]. Chromatin remodeling was observed in the context of immune response inducing a change of gene expression patterns [10]. For example, chromatin remodeling by repositioning of nucleosomes has been reported for gene promoter regions of interleukin 2 (*IL2*) [11] and colony stimulating factor 2 (*CSF2* aka *GMCSF*) [12]. Nucleosomes are 147 base pairs long DNA sequences wrapped around octamers of histone proteins [13]. They are usually separated by 10–50 bp linker sequences [14], cover 70–95% of the DNA, and are regularly spaced along chromosomes except for some, relatively rare, linkers that are much longer than 50 bp [7], [15]. In the following study, we consider linkers with a length of at least 100 bp and will refer to them as *long nucleosome-free regions (LNFRs)*. We focus on these LNFRs because they allow for a reliable detection of chromatin remodeling in terms of nucleosome repositioning, which comes down to detecting nucleosomes in regions that were nucleosome-free or to detecting LNFRs at positions which were occupied by nucleosomes in another cell state.

It can be assumed that chromatin remodeling is governed by the DNA sequence patterns, as nucleosome positions are also largely determined by them [16], [17]. Nucleosome positioning patterns are well studied. It was found that the most indicative pattern for nucleosomes are dinucleotides occurring in a 10-bp periodicity where AA/AT/TT alternates with GC [9], [15], [16], [18]–[22]. This dinucleotide pattern favors sharp bending of the DNA helical repeat every 10 bp, where the DNA sugar-phosphate backbone alternatingly faces towards the histones and away from them. Beside this periodic pattern, also local patterns, i.e. motifs, have been identified which can be sub-divided into nucleosome-favoring and nucleosome-repelling patterns. Examples of the former are the 3-mer CCA [23], 4-mers like CTAG, TAGA, TCTA [19], and the 5-mer CGCGC [7]. Nucleosome-repelling patterns specifically indicate LNFRs which are our basis for detecting chromatin remodeling in terms of nucleosome repositioning. Kaplan et al. found AAAAA and ATATA as most prominent LNFR patterns, and, more generally, identified poly(dA-dT) as LNFR indicators [24], which is in accordance with the findings of others [3], [7], [15], [21], [22], [25]–[27]. Field et al. extracted 5-mer LNFR patterns like ATATA, TAAAA [7], while Peckham et al. reported the 3-mers ATA, TAA, and AAA and the 4-mers AATA, ATAA, and AAAA [23]. In summary, the most indicative LNFR patterns are AT-rich and especially contain long A and T tracts.

We go beyond the detection of nucleosome positioning patterns and aim at identifying nucleosome *re-*positioning patterns. We focus on LNFRs to robustly find chromatin remodeling regions across the whole genome to gain insight in cellular genome re-organization dynamics in response to extracellular signals. Schones et al. [28] compared nucleosome positions in resting and activated T cells based on data from next generation sequencing (NGS). We perform a whole-genome re-analysis of these data to identify sequence patterns that govern nucleosome repositioning.

## Results

### Reliable Identification of LNFRs from NGS Data

Our read mapping resulted in 53.97% mapped reads (137,077,836 of 254,003,438) for resting T cells and 50.97% mapped reads (126,519,785 of 248,219,348) for activated T cells (further details about the mapping results are provided in Table S1). For both resting and activated T cells, we computed whole-genome nucleosome coverage profiles upon correction for multiple matches.

To assess the quality of the nucleosome coverage profiles, we investigated them at 5′ and 3′ ends of known transcripts (for details, see Text S1, Section 2). Figure S3 shows that the well-known +1 nucleosome and the 3′ NFR are clearly visible [7], [15], [21], [22], [29]. Moreover, we observed a high correlation of nucleosome coverage with H3 and H2A.Z occupancy (see Text S1, Section 3.2). All these results suggest a high quality of our nucleosome coverage profiles.

We obtained 47,270 and 79,092 LNFRs for activated and resting T cells, respectively (Table S2 shows how these LNFRs distribute over chromosomes 1–22). The average lengths of LNFRs are 154 bp for resting and 150 bp for activated T cells. Although FDR computations reveal that there might be a small proportion of false LNFRs (Text S1, Section 3.1), statistical analyses of LNFRs suggest a high quality of our LNFR sets (Text S1, Section 3.2). In particular, we analyzed overlaps of resting and activated LNFRs and overlaps of our LNFRs with LNFRs identified from an independent high-coverage nucleosome data set by Valouev et al. [9] (see Figure S6 for these LNFRs' length and GC content distributions). ChIP-seq data and conservation analysis provided further confirmation (Text S1, Section 3.2).

### LNFRs are Either AT-rich or GC-rich

**Figure 1:**
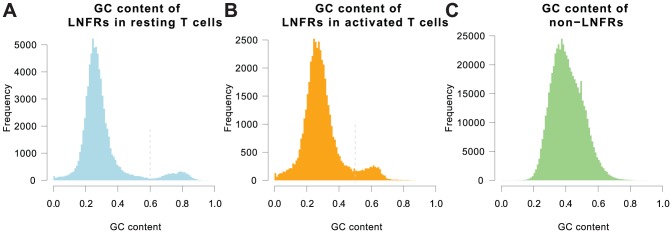
GC content distributions of LNFRs versus random fragments from the human genome. GC content distributions of LNFRs in resting T cells (**A**) and activated T cells (**B**) compared to the GC content distribution of fragments drawn randomly from the human genome (**C**). For both resting and activated T cells, the LNFRs are divided into two groups, an AT-rich and a GC-rich one. For resting T cells, the two groups are very clearly separated by a GC content threshold of about 60%, while this threshold is at 50% for activated T cells.

As mentioned in the introduction, motifs indicative for nucleosome-disfavoring sequences, and therefore also for LNFRs, were found to be mainly AT-rich, correspondingly, GC-poor. Surprisingly, the LNFRs we detected are divided into two clearly separable groups. The majority of LNFRs are AT-rich, whereas we also found a non-negligible proportion of GC-rich LNFRs. Interestingly, only few LNFRs have an average genomic GC content (about 41% in the human genome). Figure shows the GC content distributions for LNFRs in resting T cells (Figure 1A) and activated T cells (Figure 1B) in comparison to the GC content distribution of fragments drawn randomly from the human genome (Figure 1C). The GC content characteristics of LNFRs contrast strongly to those of the average genome, while the GC content distributions of LNFRs in resting and activated T cells appear to be similar, yet with some differences. In resting T cells, the two groups are very clearly separated by a GC content threshold at about 60%. The AT-rich group contains about 93% of the LNFRs and has an average GC content of 27%, whereas the GC-rich group (correspondingly, 7% or 5,403 sequences) has an average GC content of 76%. In activated T cells, the two groups are not as clearly separated, while there is still a clear trough at a GC content of around 50%. If we adopt a 50% threshold, the AT-rich group amounts to 92% of the sequences and has an average GC content of 27%, where the GC-rich group (8% or 3,922 sequences) has an average GC content of 60%.

Our observation of GC-rich LNFRs is not the result of biotechnological biases, even the opposite is the case: (1) The well-known GC bias of the Illumina Solexa technology that the read coverage is elevated in GC-rich intervals [30], even fortifies our finding of GC-rich LNFRs: since GC-rich fragments tend to be overrepresented in the set of short reads, it is likely to overestimate nucleosome occupancy in GC-rich regions, thus, to underestimate the occurrence of GC-rich nucleosome-free regions. (2) Enzymatic biases do not explain the unexpectedly prominent occurrence of GC-rich LNFRs either: Fan et al. reported the MNase cleavage site to be biased most strongly toward the dinucleotide TA [31], with AT, AA and TT being preferred sites too [31]. Hence, AT-rich nucleosome-free regions are slightly more likely to be digested by the enzyme than GC-rich ones (see Text S1, Section 1.3, and Figure S2). Correspondingly, GC-rich nucleosome free regions (LNFRs) are slightly more likely to remain undetected than AT-rich ones.

### LNFRs Outside Promoters Facilitate Genome-wide Analysis of Chromatin Remodeling

Previous analyses were mainly focused on nucleosomes and nucleosome-free regions in gene promoters [7], [15], [21], [22], [29]. Our LNFR extraction was done without any restriction to promoter regions and the majority of the LNFRs we extracted do not appear inside promoter regions (see Figure S7 and Tables S3 and S4 for details). In resting T cells, only 12.7% of the LNFRs (10,063 of 79,092) overlap with promoter regions. In activated T cells, this percentage amounts to 11.7% (5,535 of 47,270). However, LNFRs are still enriched in promoter regions (see Text S1, Section 3.3, and Tables S3 and S4).

In summary, the majority of LNFRs appear outside of promoter regions. Thus, our LNFR data facilitate a genome-wide analysis of chromatin remodeling.

### GC-rich LNFRs Exhibit a Stronger Remodeling Tendency

**Figure 2:**
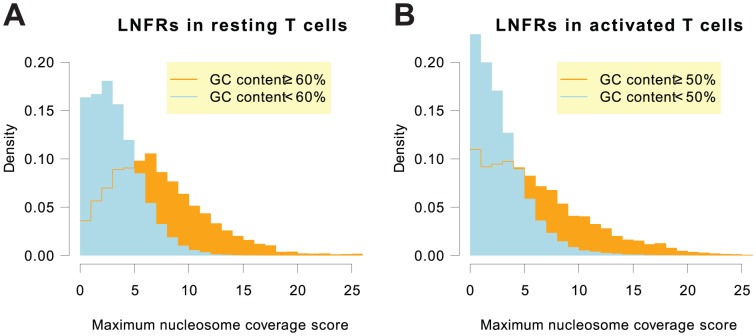
Maximum nucleosome occupancy scores over masked LNFRs. Panel **A** shows a histogram of maximum nucleosome occupancy scores in activated T cells over masked (i.e. without the first and the last 25 bp) LNFRs in resting state. Panel **B** shows a histogram of maximum nucleosome occupancy scores in resting T cells over masked LNFRs in activated state. AT-rich LNFRs generally exhibit lower nucleosome coverage than GC-rich LNFRs, which indicates that GC-rich LNFRs show a stronger remodeling tendency than AT-rich LNFRs.

We found remodeling to occur in 75% of the LNFRs in resting T cells. If we consider AT-rich and GC-rich LNFRs separately, an interesting difference is uncovered in resting T cells: 92% of GC-rich LNFRs, but only 74% of the AT-rich LNFRs, show remodeling. This observation is not as prominent in activated T cells: 63% of all, 62% of AT-rich, and 82% of GC-rich LNFRs exhibit remodeling. So, in both cell states, GC-rich LNFRs possess a stronger remodeling tendency than AT-rich LNFRs. Figure 2 shows the distributions of maximum nucleosome coverages in LNFRs measured on the respective other cell state. It is obvious that, for both cell states, AT-rich LNFRs generally have a lower nucleosome coverage in the respective other cell state than GC-rich LNFRs. Thus, GC-rich LNFRs show a stronger remodeling tendency than AT-rich LNFRs, regardless of the detection threshold.

We compared gene expression measurements of the two cell lines to investigate possible changes by nucleosome remodeling. To this end, we analyzed the gene expression data released by Schones et al. [28]. It turned out that genes have significantly lower expression levels in activated cells if their promoter regions contain an LNFR in resting T cells that is occupied by a nucleosome in activated T cells (see Text S1, Section 4.1, and Table S5). This confirms that the positioning of a nucleosome in an otherwise nucleosome-free region of a gene's promoter region tends to down-regulate this gene.

The high quality of our detection of remodeled LNFRs can be validated by ChIP-seq data for the CCCTC-binding factor (CTCF), a protein that is known to bind next to well-positioned nucleosomes [32]: the proportion of GC-rich LNFRs of resting T cells that overlap with a CTCF binding site is approximately two times as large for non-remodeled LNFRs (7%) as for remodeled LNFRs (3.6%). If all GC-rich LNFRs are considered, this difference is significant (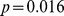 according to Fisher's exact test). If only non-promoter LNFRs are considered, the two-fold enrichment persists (2.9% vs. 6.2%), but, due to small sample numbers, it is no longer significant (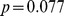 according to Fisher's exact test). In any case, it appears plausible that a protein that is associated with well-positioned nucleosomes favors LNFRs in which no nucleosome remodeling takes place.

### Remodeling Tendency is Associated With DNA Sequence Patterns

In order to extract nucleotide patterns that are specific to DNA loci where chromatin is remodeled via nucleosome repositioning, we used support vector machines (SVMs) in combination with the spectrum kernel. We applied SVMs to the following four data sets of remodeling LNFRs outside promoter regions: we split these LNFRs according to the cell state they stem from and, simultaneously, according to whether they are AT- or GC-rich. Each of the four sets of remodeled LNFRs was complemented by a set of negative sequences in order to derive SVM classifiers that can distinguish between sequences that are involved in nucleosome repositioning and other sequences (see Text S1, Section 5.1). Table 1 shows the classification accuracies for different choices of the spectrum kernel's sub-sequence length parameter 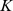.

**Table 1:** Classification performance.

	resting		activated	
*K*	AT-rich	GC-rich	AT-rich	GC-rich
1	56.1%	59.6%	50.3%	56.0%
2	59.7%	66.3%	58.8%	61.8%
3	60.3%	66.6%	59.9%	65.1%
4	60.8%	66.5%	60.4%	66.6%
5	61.7%	68.6%	60.7%	66.6%
6	61.8%	67.3%	60.9%	66.8%
7	61.6%	66.7%	60.3%	64.5%
8	61.3%	67.1%	60.0%	64.0%
9	61.4%	66.9%	59.7%	64.0%

The percentages are two-fold cross validation accuracies for the classification of LNFRs showing remodeling versus randomly selected non-LNFR DNA sequences from the human genome. Altogether four sets of remodeled LNFRs were considered: GC-rich and AT-rich remodeled LNFRs, both for resting and activated T cells. Each row corresponds to one choice of 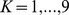, the sub-sequence length parameter of the spectrum kernel. GC-rich remodeling LNFRs (third and fifth column) can be classified with higher accuracy than AT-rich remodeling LNFRs (second and fourth column). The highest accuracy for GC-rich remodeling LNFRs in resting T cells is 68.6% and is achieved for 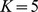.

We found that LNFRs associated with remodeling can be distinguished from randomly selected non-LNFR nucleotide sequences with high accuracy on the basis of their nucleotide patterns only. This result indicates that nucleosome repositioning is associated with DNA sequence patterns. Since we have equated the two classes in terms of their GC content, the classifiers rely on the appearance of certain non-trivial DNA sequence patterns that are not related to mere GC content.

### Remodeling Patterns are More Specific and More Prominent in Resting Than in Activated T Cells

**Figure 3:**
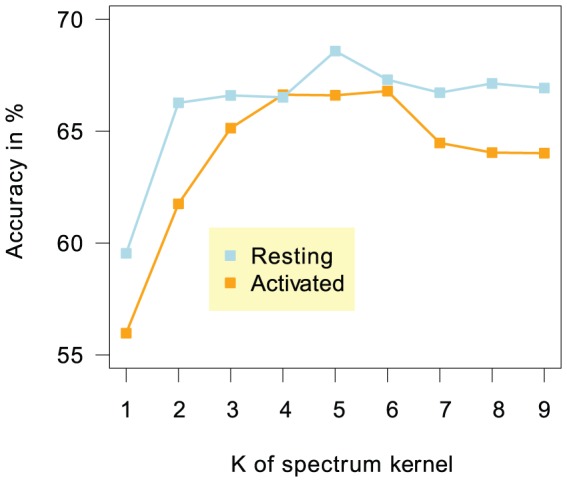
Classification performance versus sub-sequence length. Two-fold cross validation accuracies for the classification of GC-rich LNFRs showing remodeling versus randomly selected non-LNFR sequences for different choices of 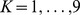, the sub-sequence length parameter of the spectrum kernel. For resting T cells, the accuracy peaks at 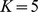, while 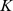
's between 4 and 6 are best for classifying GC-rich remodeling LNFRs in activated T cells.

The results in Table 1, in particular, show that the accuracy in classifying GC-rich remodeling LNFRs versus randomly selected DNA sequences is significantly higher than in classifying AT-rich remodeling LNFRs (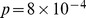 according to a one-sided Kolmogorov-Smirnov test). In resting T cells, both GC-rich and AT-rich remodeled LNFRs can be better distinguished from randomly selected DNA sequences than in activated T cells. Figure 3 shows a plot of accuracies versus the sub-sequence length parameter 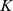 of the spectrum kernel. The plot reveals that remodeled GC-rich remodeled LNFRs can be identified from their DNA sequences with higher accuracy for resting T cells than for activated T cells. Moreover, the drop of accuracies with increasing 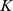 is more severe for activated than for resting T cells. This suggests that the sequence patterns characterizing GC-rich remodeled LNFRs of resting T cells are more specific and more prominent than for activated T cells.

That GC-rich remodeled LNFRs of T cells can be characterized best by an SVM with 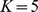 does not mean that the patterns that are most typical for remodeled GC-rich LNFRs of resting T cells are actually 5 bases long. The sub-sequence length of 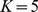 just balances underfitting and overfitting in the best way by exploiting overlaps of indicative patterns. However, the decrease of accuracies for 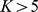 is moderate if we take into account that the dimensionality of the feature space grows exponentially with 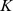. So it is plausible that the patterns that are most typical for remodeled LNFRs are actually longer. In order to identify those indicative patterns independently of the choice of 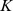, we implemented a procedure that computes prediction profiles for all remodeled LNFRs (see Figure S8 for an example), then extracts regions of interest, and finally uses a motif finder to identify motifs commonly occurring in these regions of interest (see **Materials and Methods** below and Text S1, Section 5). That this procedure generalizes to previously unseen data has been verified as well (see Table S6 and Text S1, Section 5.3).

**Figure 4:**
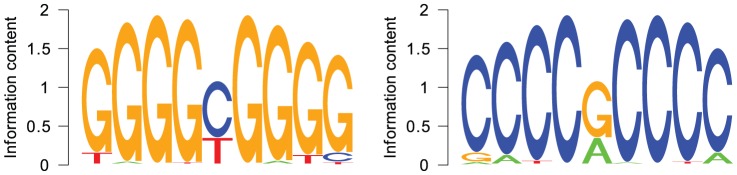
Sequence logo of remodeling pattern for both DNA strands. The pattern was identified by applying MEME to the regions of interest obtained from the prediction profiles computed by the SVM with the spectrum kernel with 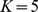.

For the best choice in terms of cross validation accuracy, 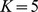, we identified 166 regions of interest for the entire set of remodeled GC-rich LNFRs of resting T cells. The motif finder software MEME [33], [34] found exactly one pattern to be typical for those regions of interest, the sequence logos of which are shown in Figure 4. According to MEME, the regular expressions describing this pattern are GGGG[CT]GGGG and CCCC[GA]CCCC, respectively. Figure S9 shows regular expressions and sequence logos of the patterns identified to be typical for the regions of interest determined from the support vector machines trained with 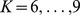. These patterns are slightly longer, but they are similar to the pattern GGGG[CT]GGGG/CCCC[GA]CCCC in the sense that they all contain a single C or T position that occurs in a longer G tract.

The pattern GGGG[CT]GGGG/CCCC[GA]CCCC occurs in 36.7% of positive sequences (GC-rich remodeled resting LNFRs outside promoters) and in 16.7% of negative sequences (random non-LNFR sequences with same length and GC content distribution). So the pattern is indeed enriched in positive sequences (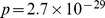 according to Fisher's exact test). The pattern GGGG[CT]GGGG/CCCC[GA]CCCC occurs in 23.7% of GC-rich *non-*remodeled resting LNFRs outside promoters (compare with Table 2). This is a significantly lower percentage than in remodeled LNFRs (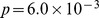 according to Fisher's exact test, see also Table 2). If we compare remodeled vs. non-remodeled GC-rich resting LNFRs regardless of whether they are inside or outside promoters, the enrichment of the pattern GGGG[CT]GGGG/CCCC[GA]CCCC in remodeled sequences is also significant (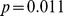 according to Fisher's exact test; see Table 2 for exact details). The significant enrichment in remodeled versus non-remodeled LNFRs makes clear that the pattern GGGG[CT]GGGG/CCCC[GA]CCCC is not only indicative for LNFRs, but indeed *indicative for nucleosome remodeling in resting T cells*.

**Table 2:** Occurrences of certain patterns in remodeled vs. non-remodeled GC-rich LNFRs of resting T cells.

	outside promoters			inside and outside promoters		
	remodeled	non-rem.	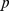 -value	remodeled	non-rem.	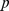 -value
	(total: 1212)	(total: 97)	(Fisher t.)	(total: 4949)	(total: 199)	(Fisher t.)
GGGG[CT]GGGG	36.7%	23.7%	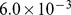	39.9%	31.7%	0.011
CCCC[GA]CCCC						
GGGGCGGGG	25.5%	14.4%	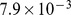	31.7%	23.1%	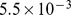
CCCCGCCCC						
GGGGTGGGG	16.8%	11.3%	0.10	13.2%	11.6%	0.28
CCCCACCCC						
G-quadruplex pattern	33.8%	15.5%	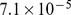	36.4%	16.6%	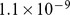

Columns 2 and 3 provide the percentages of GC-rich resting LNFRs outside promoters in which the patterns occur. Column 4 provides the 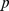-value of Fisher's exact test for enrichment of the patterns in remodeled LNFRs. Columns 5–7 are analogous to columns 2–4, but the percentages and 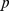-values are computed for all GC-rich resting LNFRs, both inside and outside promoters. The percentages in the first row are not the exact sums of percentages in rows 2 and 3 because there are LNFR sequences that contain both patterns GGGGCGGGG/CCCCGCCCC and GGGGTGGGG/CCCCACCCC.

**Figure 5:**
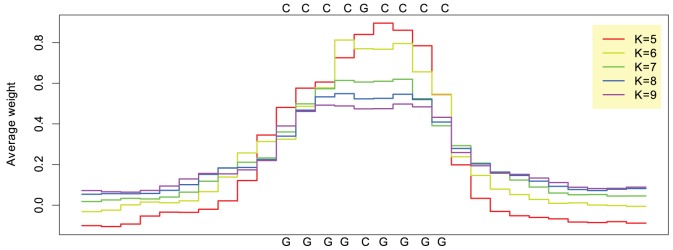
Average prediction profiles around occurrences of the pattern GGGGCGGGG/CCCCGCCCC. Each curve corresponds to the average prediction profiles around the occurrences of the pattern in GC-rich remodeled LNFRs of resting T cells. The different curves correspond to different choices of 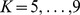.

The pattern GGGG[CT]GGGG/CCCC[GA]CCCC actually consists of two mutually exclusive pattern variants, GGGGCGGGG/CCCCGCCCC and GGGGTGGGG/CCCCACCCC. Studying them separately provides an even clearer picture: GGGGCGGGG/CCCCGCCCC occurs in 25.5% of positive sequences (GC-rich remodeled resting LNFRs outside promoters) and in 12% of negative sequences (random non-LNFR sequences with same length and GC content distribution) — which is significant with 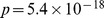 according to Fisher's exact test. The pattern GGGGTGGGG/CCCCACCCC occurs in 16.8% of positive sequences and in 6.4% of negative sequences — which is also significant with 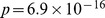 according to Fisher's exact test. This confirms that both patterns are typical for GC-rich remodeled resting LNFRs outside promoters, as opposed to the corresponding negative set of non-LNFR sequences. The more interesting question is whether both are actually remodeling patterns. As shown in Table 2, GGGGCGGGG/CCCCGCCCC occurs significantly more often in remodeled LNFRs than in non-remodeled LNFRs (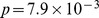 and 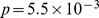, respectively). The pattern GGGGTGGGG/CCCCACCCC also occurs more often in remodeled than in non-remodeled LNFRs, but this difference is not significant. This suggests that GGGGTGGGG/CCCCACCCC is rather a general LNFR pattern than a remodeling pattern, whereas GGGGCGGGG/CCCCGCCCC is indeed a *nucleosome remodeling pattern*. Figure 5 plots the average prediction profiles around all occurrences of this pattern in GC-rich remodeled LNFRs of resting T cells. This plot confirms that all SVMs with different choices of the sub-sequence length parameter 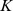 agree that the pattern is indicative for the positive class (GC-rich remodeled resting LNFRs outside promoters).

### CpG Islands are Associated with Nucleosome Remodeling Via CpG-island-binding Proteins

To attribute biological functions to the nucleotide patterns that are most characteristic for nucleosome repositioning in GC-rich LNFRs, we searched the JASPAR database of transcription factor binding profiles [35] for matches with the pattern GGGGCGGGG/CCCCGCCCC. The only reasonable match among proteins of higher organisms was the Sp1 transcription factor (Specificity Protein 1) which has already been verified to be involved in chromatin remodeling in the promoter region of the *GMCSF* gene [36]. However, the analysis of Sp1 ChIP-seq data from the ENCODE project [37] has not shown any enrichment of Sp1 binding sites in remodeled sequences; even the opposite is true: Sp1 binding sites occur more frequently in non-remodeled sequences (though not significantly; see Table 3 for details). The Sp1 ChIP-seq data have been obtained from a different cell type (see Text S1, Section 3.2, for details), so we cannot definitely rule out the involvement of Sp1 in genome-wide chromatin remodeling, but these data do not supply any evidence in favor of this assumption either. Most other matches obtained from the JASPAR database were zinc-finger proteins, but all of them were proteins of very distant organisms, such as, yeast.

**Table 3:** Overlaps with CpG islands and binding site peaks of remodeled vs. non-remodeled GC-rich LNFRs of resting T cells.

	outside promoters			inside and outside promoters		
	remodeled	non-rem.	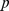 -value	remodeled	non-rem.	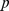 -value
	(total: 1212)	(total: 97)	(Fisher t.)	(total: 4949)	(total: 199)	(Fisher t.)
CpG island	58.2%	25.8%	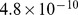	77.2%	40.2%	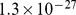
Sp1 peak	8.8%	10.3%	*0.36*	19.5%	21.1%	*0.31*
KDM2A peak	45.7%	11.3%	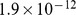	72.8%	28.6%	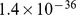
CFP1 peak	21.4%	6.2%	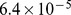	50.9%	25.6%	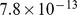
Pol II peak (A)	13.0%	8.3%	0.11	34.5%	22.6%	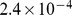
Pol II peak (B)	3.05%	3.09%	*0.58*	10.4%	12.6%	*0.20*

Columns 2 and 3 provide the percentages of GC-rich resting LNFRs outside promoters that overlap with CpG islands or the binding site peaks under consideration. Column 4 provides the 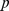-value of Fisher's exact test for enrichment of overlaps in remodeled LNFRs, except for value typeset in italics, which correspond to 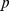-values of Fisher's exact test for enrichment of overlaps in non-remodeled sequences. Columns 5–7 are analogous to columns 2–4, but the all percentages and 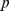-values are computed for all GC-rich LNFRs, no matter whether inside or outside promoters. The row “Pol II (A)” refers to the Pol II ChIP-seq data set published by Barski et al. [46], while “Pol II (B)” refers to the Pol II ChIP-seq data published by Schones et al. [28].

**Figure 6:**
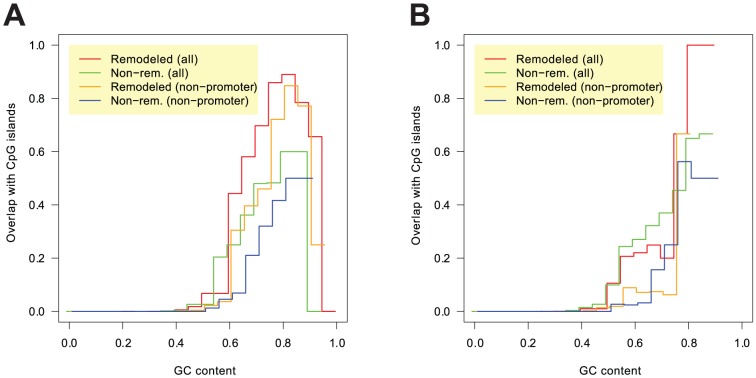
Proportion of LNFRs overlapping with CpG islands plotted versus the LNFRs' GC content. Each curve plots the proportion of LNFRs overlapping with CpG islands in relation to the GC content of the considered LNFRs. The plot in panel **A** shows data for LNFRs of resting T cells, while the plot in panel **B** shows data for LNFRs of activated T cells. For resting T cells, a clear difference between remodeled and non-remodeled LNFRs is visible, both if we consider all LNFRs and if we restrict to non-promoter LNFRs. The proportions of overlaps of LNFRs of activated T cells with CpG islands are generally lower and no clear difference is visible between remodeled and non-remodeled LNFRs.

The pattern GGGGCGGGG/CCCCGCCCC contains a CpG site and we have further found out that this pattern or similar patterns (e.g. G tracts interrupted by a C, resp. C tracts interrupted by a G) usually occur multiple times in remodeled GC-rich LNFRs (see Figure S8 for a not necessarily representative, but illustrative, example). This fact particularly hints at CpG islands, that is, GC-rich regions with an elevated frequency of usually unmethylated CpG sites [38]. As expected, CpG islands are 41 times enriched of the pattern GGGGCGGGG/CCCCGCCCC: of 47,773 occurrences of this pattern in chromosomes 1–22 of hg18, 14,770 are inside annotated CpG islands. If the patterns and CpG islands were independent (the null hypothesis of the test), the expected number of patterns in CpG islands would be 361. This difference is, of course, highly significant (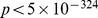 according to a binomial test). However, this could partly or entirely be caused by the fact that pattern frequencies and overlaps with CpG islands are both strongly related to the GC content. The more interesting question is whether any significant difference can be observed between remodeled and non-remodeled GC-rich LNFRs. The first row of Table 3 shows that there are significantly more overlaps of CpG islands with remodeled LNFRs than with non-remodeled LNFRs of resting T cells. There is approximately a two-fold enrichment in remodeled LNFRs, both if we restrict to GC-rich non-promoter LNFRs and if we consider all GC-rich LNFRs. As expected, the overall ratios of overlaps with CpG islands are much larger if we include promoter LNFRs. We also analyzed overlaps of remodeled and non-remodeled LNFRs with CpG islands for different levels of GC content in order to find out whether the observed differences are a mere effect of GC content. The plot in Figure 6A rules out that this is only a GC content effect: for almost every GC content level, the proportion of overlaps of non-remodeled LNFRs with CpG islands is considerably below the proportion of overlaps of remodeled LNFRs with CpG islands. Figure 6B demonstrates that the proportions of overlaps of LNFRs of activated T cells with CpG islands are generally lower and no clear difference between remodeled and non-remodeled LNFRs can be seen. All these findings fit to the results obtained for methylation data (see Figure S10 and Text S1, Section 6).

Now that we have identified a strong association with CpG islands, the question is how CpG islands are related to nucleosome remodeling. It is known that proteins with ZF-CXXC domains bind to unmethylated CpG sites in CpG islands [39] (note that our search in the JASPAR database already hinted at zinc finger proteins). CXXC Finger Protein 1 (CFP1) and Lysine-specific demethylase 2A (KDM2A) are two ZF-CXXC proteins and have previously been reported to participate in chromatin remodeling by binding to nucleosome-free CpG sites in CpG islands [40]–[44]. In order to study the association between CFP1 and KDM2A binding sites and nucleosome remodeling, we analyzed CFP1 and KDM2A ChIP-seq data. Since human data are not available for these two proteins, we resorted to mouse data and mapped the results to the human genome. We found KDM2A binding site peaks to overlap with 45.7% of remodeled GC-rich non-promoter LNFRs of resting T cells, but only with 11.3% of non-remodeled GC-rich non-promoter LNFRs or resting T cells, which is a highly significant difference (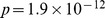 according to Fisher's exact test; see also Table 3). If we also consider promoter LNFRs, the difference is even more significant: 72.8% of all remodeled GC-rich LNFRs of resting T cells overlap with KDM2A binding site peaks, as opposed to 28.6% of all non-remodeled GC-rich LNFRs of resting T cells (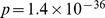 according to Fisher's exact test). For CFP1 binding site peaks, a similar picture is obtained: the differences are less pronounced, but still highly significant (see Table 3).

Blackledge and Klose [45] have described pathways in which CFP1 and KDM2A participate together with RNA polymerase II (Pol II) in regulatory functions by modifying chromatin in CpG islands. In order to verify or falsify whether this mechanism is a major cause or our findings, we analyzed two Pol II ChIP-seq data sets [28], [46]. First, in all cases but one, no significant difference of overlaps with Pol II binding site peaks occurs between remodeled and non-remodeled LNFRs. Secondly, the rates of overlaps with Pol II binding site peaks are generally much lower than for KDM2A or CFP1 (see Table 3). This difference is particularly high if we restrict to non-promoter LNFRs, in which, as expected, the occurrences of Pol II binding site peaks are quite sparse. We conclude that the mechanisms underlying the role of KDM2A and CFP1 in nucleosome remodeling of resting T cells, in particular, outside promoter regions, are not or only remotely related to Pol II.

## Discussion

We studied chromatin remodeling in terms of nucleosome repositioning by comparing nucleosome occupancy in resting and activated human T cells. Nucleosome repositioning was detected in long nucleosome-free regions (LNFRs) that were occupied by nucleosomes in the respective other cell state. An interesting observation was the fact that 67% more LNFRs were obtained for resting than for activated T cells (79,092 LNFRs in resting versus 47,270 LNFRs in activated T cells). Our explaination is that the chromatin structure of resting T cells is more deterministic to enable a fast activation. The discrepancy in LNFR numbers cannot be explained by different lengths, since the lengths of LNFRs are similarly distributed for the two cell states (see above and Figure S5).

Regardless of the cell state, we observed that GC-rich LNFRs are more likely to be subject to nucleosome repositioning than AT-rich LNFRs. That AT-rich LNFRs are less often remodeled fits well to previous findings that AT-rich patterns largely disfavor nucleosome occupancy. The pattern we identified to be characteristic for nucleosome repositioning does not have any resemblance with the patterns known to favor nucleosomes either. This indicates that nucleosome repositioning is indeed governed by highly specific sequence characteristics that are complementary to the nucleosome positioning code consisting of well-known nucleosome-favoring and -repelling patterns.

Remodeling is not restricted to promoter regions, since we found a considerable proportion of remodeled LNFRs outside promoter regions. Thus remodeling is supposed to serve for further purposes besides regulation of transcription by nucleosome shifts in the promoter region, especially around the transcription start site. We suggest that chromatin remodeling is involved in a whole 3D structure conformation change of chromatin which brings DNA regions to physical proximity that are distant with respect to linear genomic locations. Such structural changes could have dramatic effects on the cell's regulatory and transcriptomic dynamics, as there is clear evidence of a relationship between the genes' physical arrangement and gene activation [47], [48].

Our results indicate a genome-wide role of CpG islands in nucleosome remodeling, governed by proteins that favor binding to unmethylated CpG sites in CpG islands. Two representatives of this class, KDM2A and CFP1, were found to bind significantly more often to remodeled LNFRs of resting T cells than to non-remodeled LNFRs. This result, however, was obtained from ChIP-seq data of mouse cells, so the uncertainty remains whether the same results would actually be obtained for human T cells. We are strongly convinced that our results are far too significant to be the result of pure chance. Pairwise global alignments of human and mouse versions of the two proteins showed that their amino acid sequences are highly similar (see Text S1, Section 7, and Figures S11 and S12), which makes it plausible that both KDM2A and CFP1 occur structurally similarly in humans and mice and that they will bind to the same sequence patterns in human cells and in mouse cells.

An alternative explanation for GC-rich remodeled LNFRs in resting T cells might be that those sequences are involved in the formation of G-quadruplexes [49], [50]. They consist of a square arrangement of guanines that is stabilized by hydrogen bonds and a cation in the center of the square structure. The DNA pattern for a G-quadruplex is 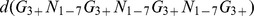, which means that four tracts of 3 or more guanines are separated by 1–7 arbitrary nucleotides, where the length of the G tracts is the same for all four tracts. G-quadruplexes have been found in vivo, occur in nucleosome-free regions, and are supposed to play regulatory roles [51]. We found an enrichment of potential G-quadruplex forming sequence patterns in GC-rich remodeled LNFRs compared to GC-rich non-remodeled LNFRs: 33.8% versus 15.5% if we restrict to non-promoter LNFRs; 36.4% versus 16.6% if we consider all GC-rich LNFRs, which is even more significant than the pattern GGGGCGGGG/CCCCGCCCC (see Table 2 for detailed figures). However, the difference in overlaps of CFP1 and KDM2A binding sites is still much more significant. Moreover, it is considered likely that G-quadruplexes are only formed at a small proportion of occurrence of G-quadruplex patterns [50].

Summarizing all results and discussions from above, we suggest a genome-wide role of CpG-islands in chromatin remodeling via ZF-CXXC proteins.

## Materials and Methods

### NGS Data and Read Mapping

Our study is based on the next generation sequencing data provided by Schones et al. [28]. The data set consists of short reads of ends of nucleosomal DNA to determine nucleosome positions in resting and activated human CD4+ T cells. Details about the data set are provided in Text S1, Section 1, complemented by Figures S1 and S2 which show the reads' GC content distribution and the nucleotide distributions at each position, respectively. The reads were mapped to hg18 (NCBI Build 36.1) using SOAP [52]. In contrast to the original mapping, we used a more liberal read mapping strategy and allowed for one mismatch or one gap. Most importantly, we did not restrict the mapping to uniquely mappable reads, but mapped each read to all its best matching positions. For detailed mapping results, see Table S1 and Text S1, Section 1. With this mapping strategy, we deliberately accept that we might detect nucleosomes wrongly in order to ensure that our detection of LNFRs is highly specific.

### Nucleosome Coverage Profiles

We computed nucleosome coverage profiles for both cell states. For each position in the genome, the coverage value corresponds to the number of reads that indicate a nucleosome at this position. Every uniquely matched read contributes a value of 1 to the coverage profile at each nucleotide it matches. Since every read only covers the first 24 bases of a nucleosome, we also incremented the coverage profile at all 126 bases downstream of the match (i.e., we extend the read to 150 bases in total; see Figures S4A and S4B for an illustration). If the read was matched via its reverse complement, this extension is done in the opposite direction. Non-unique matches are taken into account too, but the contribution of a non-unique match is chosen relative to its number of matches, e.g. a read that matches 4 positions on the genome contributes 0.25 to the coverage profile at all nucleotides it matches.

### Extraction of LNFRs

We identified LNFRs as contiguous DNA sequences with zero nucleosome coverage that are at least 100 bp long (see Figure S4C for an illustrative example).

### Database of Transcripts

We use the table “UCSC Known Genes” [53], [54] as reference data source of transcripts in the human genome. We used the version of September 29, 2011, as included in the R/Bioconductor package TxDb.Hsapiens.UCSC.hg19.knownGene and mapped its hg19 locations to the hg18 genome using the LiftOver tool [55]. This data set contains the genomic locations of 77,614 coding and non-coding transcripts, of which 70,663 are transcribed from chromosomes 1–22, where the total number of distinct transcription start sites that could be mapped to hg18 is 42,499.

### Identification of Chromatin Remodeling

We define an LNFR in one condition (resting or activated) to exhibit remodeling if we detect nucleosome occupancy in this region in the respective other condition (activated or resting). We do not consider the first 25 and the last 25 bases of each LNFR in order to avoid that minor shifts of nucleosomes are detected as remodeling. This masking is necessary because the biotechnology does not ensure precise positions of nucleosome ends. We consider an LNFR to be a remodeling locus if there is at least one base (excluding the first 25 and the last 25) that has nucleosome coverage of at least 2, i.e. at least two reads must indicate a nucleosome (Text S1, Section 3). We chose this threshold to account for possibly false nucleosome detections introduced by our liberal mapping strategy.

Furthermore, we consider an LNFR not to be a remodeling locus if no base (again excluding the first 25 and the last 25) has a nucleosome coverage of more than 1, i.e. at most one read may indicate a nucleosome in the respective other cell state.

### Microarray Data Preprocessing

The microarray data provided by Schones et al. [28] were processed using RMA [56]. Since the data set consists of only four arrays, it is not meaningful to use standard methods, such as, LIMMA [57] for determining differentially expressed genes. Instead, we used simple difference scores to evaluate the difference between expression levels in the two types of T cells (Text S1, Section 4.1).

### Identification of Remodeling Patterns Using Support Vector Machines (SVMs)

We choose SVMs [58], [59] for identifying remodeling patterns because they performed well in various biological classification tasks, for instance, in promoter and splice site detection [60]–[62] or protein fold and secondary structure prediction [63]–[65]. The discriminative approach of SVMs has the advantage that it allows for identifying sequence patterns that are specific to nucleosome repositioning loci without mingling them with nucleotide patterns that occur frequently throughout the genome. The discriminative approach, however, necessitates the construction of a set of *negative (control) sequences*, whereas the nucleotide sequences at nucleosome repositioning loci are considered as *positive samples*. We draw sequences randomly from the human genome excluding the identified LNFRs. In order to avoid obscuring our results with mere GC patterns, we further require the negative set to have the same GC content distribution as the respective positive nucleosome repositioning set (Text S1, Section 5.1). Following this procedure, we generated balanced data sets on which we applied SVMs.

The application of SVMs to sequence data requires a kernel that computes the similarity between two biological sequences. We used the well-known *spectrum kernel* [63] which, roughly speaking, computes the similarity of two sequences as the number of *K*-mers, i.e. gap-less sub-sequences of length *K*, they have in common. For each SVM that we trained using the spectrum kernel, we extracted indicative sequence patterns following a strategy similar to [66], [67] (Text S1, Section 5.2). These pattern weights facilitate the computation of prediction profiles [67] from which we can extract regions of interest, i.e. sub-sequences the SVM considers particularly typical of remodeled LNFRs (Text S1, Section 5.3). Subsequently, we applied the MEME motif finder [33], [34] to those regions of interest.

### Identification of Binding Site Patterns

We searched the JASPAR database [35] for matches between nucleosome repositioning patterns and known transcription factor binding sites. We computed the likelihood score that a pattern is a representative of the binding sites for all 1,316 transcription factors. The likelihood scores were optimized via gap-less alignments between the pattern and the frequency matrix.

### Analysis of CpG Island Data

We downloaded the UCSC Genome Browser track “cpgIslandsExt” (version as of Feb. 18, 2012) which contains 28,226 CpG islands, 26,567 of which are on chromosomes 1–22. To test for the enrichment of sequence patterns in CpG islands, we used a binomial test. For analyzing overlaps with different types of LNFRs, we used Fisher's exact test.

### Analysis of Human ChIP-seq Data

We mapped reads to hg18 (NCBI Build 36.1) using Bowtie [68]. We allowed for two mismatches and considered only unique matches as recommended, e.g., by [69]. ChIP-seq peaks were determined using the recent R/Bioconductor package BayesPeak [70], [71]. Tests for enrichment of peaks in genomic regions were performed using a binomial test and tests for overlaps of peaks with distinct groups of genomic regions (e.g., remodeled vs. non-remodeled LNFRs) were performed using Fisher's exact test.

### Analysis of CFP1 and KDM2A ChIP-seq Data

We considered the CFP1 ChIP-seq data set of Thomson et al. [40] (SRA Accession SRX017083) and the KDM2A ChIP-seq data set of Blackledge et al. [41] (SRA Accession SRX017108). Both data sets have been obtained from mouse cells, the former from brain cells, the latter from embryonic stem cells. The two data sets were processed with the same analysis pipeline as the human ChIP-seq data sets, except that the mapping and the peak analysis were performed on the mouse mm10 genome (Genome Reference Consortium GRCm38). The final peaks were then mapped from mm10 to hg19 (Genome Reference Consortium GRCh37) and further to hg18 using the UCSC Genome Browser LiftOver tool [55].

## Supporting Information

Text S1 **Additional analyses and further details on methods and materials.**
(PDF)

Figure S1 **GC content of raw sequencing reads.** The average GC content of raw sequence reads is significantly higher for activated (48%) than for resting T cells (44%).
(PDF)

Figure S2 **Raw sequence reads are strongly biased to adenine at the first position of the read.** Panel **A**: the average genomic content of nucleotides (dark blue bar labeled “hg18”) is compared to the average content at positions 1–8 of the sequencing reads (light blue bars labeled “pos1”, “pos2”, etc.). Panel **B**: average genomic nucleotide content versus average nucleotide content at positions 1–24 of reads obtained for resting T cells; Panel **B**: anologous to panel **B** for activated T cells.
(PDF)

Figure S3 **Average nucleosome coverage profiles around the transcription start sites (TSS) and 3′ ends of transcripts.** The well-known +1 nucleosome and the 3′ NFR are clearly visible.
(PDF)

Figure S4 **Illustration of computation of nucleosome coverage profiles and LNFR extraction.**
(PDF)

Figure S5 **Distributions of LNFR lengths for resting and activated T cells.** The length distributions are very similar. Furthermore, no sequencing or biotechnology artifact is visible.
(PDF)

Figure S6 **Characteristics of LNFRs extracted from Valouev et al. 's high-coverage data.** Panel **A** shows the distribution of lengths and panel **B** shows the GC content distribution.
(PDF)

Figure S7 **Proportions of LNFRs overlapping with promoter regions [-10 kbp, +1 kbp] (red graphs) versus their GC content.** Panel **A**: data for LNFRs of resting T cells. Panel **B**: data for LNFRs of activated T cells. The histograms provide the numbers of LNFRs in dependence of GC content.
(PDF)

Figure S8
**SVM prediction profiles for an exemplary sub-region of an LNFR (pos. 131,892,094–131,892,186 of chromosome 10 in hg18).** The larger 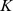, the smoother the prediction profile. The five profiles agree on the fact that the region marked by the gray background is typical for the positive class (remodeled GC-rich LNFRs).(PDF)

Figure S9
**Patterns indicative for remodeled LNFRs obtained from SVM predictions profiles for K's ranging from 6 to 9.** The pattern for 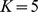 is shown in Figure 4.(PDF)

Figure S10 **Proportion of LNFRs overlapping with methylation sites plotted versus the LNFRs' GC content.** Each curve plots the proportion of LNFRs overlapping with methylation sites in relation to the GC content of the considered LNFRs. For resting T cells (panel **A**), at a GC content of around 80%, the ratio of methylated non-remodeled LNFRs is indeed much higher than the proportion of methylated remodeled LNFRs. Exactly at this GC content, the largest difference in overlaps with CpG islands occurs (compare with Figure 6). For activated T cells (panel **B**), the differences are not so evident, but it should be pointed out that, for a GC content up to 75%, the rates of methylated LNFRs are generally lower than for resting T cells.
(PDF)

Figure S11 **Global alignment of human and mouse KDM2A sequences.** There is one single-residue gap. Of the aligned residues, 1,144 are similar (98.5%) and 1,129 are identical (97.2%). The CXXC domain is free of any mismatches.
(PDF)

Figure S12 **Global alignment of human and mouse CFP1 sequences.** There is one four-residue indel. Of the aligned residues, 638 are similar (96.7%) and 635 are even identical (96.2%). The CXXC domain is free of any mismatches.
(PDF)

Table S1 **Statistics of mapping short reads using SOAP for resting and activated T cells.**
(PDF)

Table S2 **Numbers of extracted LNFRs in all autosomal chromosomes in the human genome.**
(PDF)

Table S3 **Numbers of LNFRs overlapping with promoter regions [−1 kbp, +1 kbp].**
(PDF)

Table S4 **Numbers of LNFRs overlapping with promoter regions [−100 bp, +100 bp].**
(PDF)

Table S5 *p* **-values of tests for differential expression of remodeled genes versus non-remodeled genes.** All four tests indicate significance for all three scores (details to be found in Text S1, Section 4.1).
(PDF)

Table S6 **Results** **of cross validation analysis of pattern extraction procedure.** In the majority of cases, the motif extraction procedure produces significant motifs and, in case they are significantly enriched on the training set, they are also significantly enriched on the test fold.
(PDF)
