# Supplementary material for: Genome-Wide Chromatin Remodeling Identified at GC-Rich Long Nucleosome-Free Regions
Source: PLoS One. 2012 Nov 5;7(11):e47924. doi: 10.1371/journal.pone.0047924 (PMC3489898; doi:10.1371/journal.pone.0047924)
Supplement: Text S1 — Additional analyses and further details on methods and materials. (PDF) [file pone.0047924.s001.pdf]

Genome-Wide Chromatin Remodeling Identified at GC-Rich Long Nucleosome-Free Regions

— Supporting Information —

Karin Schwarzbauer, Ulrich Bodenhofer, and Sepp Hochreiter
Institute of Bioinformatics, Johannes Kepler University, 4040 Linz, Austria

Institute of Bioinformatics
Johannes Kepler University Linz
A-4040 Linz, Austria

Tel. +43 732 2468 8880
Fax +43 732 2468 9511
http://www.bioinf.jku.at

1 NGS Data and Read Mapping

The raw sequencing data of Schones et al. [1] stem from human T cells in two different conditions: resting and activated (SRA Accessions SRX000168 and SRX000164, respectively). For each of these data sets, we obtained almost 300 million reads of length 24–25 bp which are associated with ends of nucleosomes. Upon merging duplicates, the total number of distinct reads was around 250 millions for both data sets. Using the SOAP software [2], we mapped these reads to the human genome version hg18 (NCBI Build 36.1). For the mapping, we allowed for one mismatch or one gap with a maximum length of 3 bp, which has been reported by Dohm et al. as being optimal for trading read errors off against false positives [3]. Most importantly, we did not restrict the mapping to uniquely mappable reads, but mapped each read to all its best matching positions. For short reads that can be mapped without a mismatch, all positions of perfect matches were reported, but no positions where a mismatch would appear. If the read cannot be mapped perfectly, but only with one mismatch, then all positions where the read can be mapped with one mismatch are reported, and so on. Thereby, a match involving a mismatch is viewed to be better than a match inserting a gap. With this strategy, we might wrongly detect nucleosomes, but the detection of LNFRs is highly specific.

1.1 Mapping Results

The mapping resulted in 137,077,836 (53.97%) mapped reads out of 254,003,438 distinct reads for resting T cells and 126,519,785 (50.97%) mapped reads out of 248,219,348 distinct reads for activated T cells. The numbers of reads with perfect matches, matches with a mismatch and matches with a gap are shown in Table S1. Only about 44% of the reads could be mapped uniquely, which shows the high percentage of reads that were not taken into account in the original mapping performed by Schones et al. [1].

1.2 GC Content of Raw Sequencing Reads

We computed the GC content of the raw sequencing reads for both resting and activated T cells, where we excluded all short reads containing gaps (see Figure S1 for histograms). The average GC content for activated T cells is 48% and 44% for resting T cells. This difference is significant according to a one-sided t-test ($p < 5 \times 10^{-324}$).

1.3 Biases/Characteristics Detected in Raw Sequencing Reads

We further computed the frequencies of nucleotides A, C, G and T at each position of the raw sequencing reads. The analysis reveals a strong bias towards adenine at the first position of the short reads (see Figure S2A), whereas guanine and cytosine appear much less frequently at this position. The same analysis applied to sequencing data provided by the 1000 Genomes Project [4] did not reveal such a strong deviation from the average genomic nucleotide distribution, which suggests that the bias mainly stems from the fragmentation process done by digestion of chromatin using MNase.

Figures S2B and S2C display the proportions of each nucleotide along the whole short read (24 bases) for resting and activated T cells, respectively. Again it can be seen that the GC content

is significantly higher for activated than for resting T cells. Furthermore, the bias of higher read coverage in GC-rich intervals [3] is obvious at activated T cells and to a lesser extent at resting T cells. Besides potential biases due to the NGS technology, the enzyme used for digesting the DNA to mononucleosomal fragments introduces biases. The enzyme's most preferred cutting site is the dinucleotide `TA`, followed by `AT`, `AA` and `TT` [5], which is strongly reflected in our data.

2 Nucleosome Coverage Around Transcription Start Sites and 3' Ends of Transcripts

To assess the quality of the nucleosome coverage profiles, we investigated nucleosome coverage at the ends of known transcripts. Figure S3A depicts the average nucleosome coverage profiles in the region 3 kbp around the transcription start sites (TSS) of known transcripts. Despite our liberal mapping strategy, the profiles show a trough immediately upstream of the TSS, followed by a peak shortly downstream of the TSS, indicating a well positioned nucleosome, which is well known as the +1 nucleosome [6, 7, 8, 9, 10]. The profiles further indicate that the peak corresponding to the +1 nucleosome is more pronounced in activated T cells. Figure S3B depicts the average nucleosome coverage profiles in the region 3 kbp around the 3' ends of known transcripts. The profiles show a pronounced trough at the 3' end, which corresponds to the known 3' NFR [7]. Thus, we were able to confirm previous findings, suggesting a high quality of our nucleosome coverage profiles.

3 Extraction of LNFRs from the Nucleosome Coverage Profiles

Using the nucleosome coverage profiles, as explained in the main manuscript and illustrated in Figure S4, we identified LNFRs as contiguous DNA sequences with zero nucleosome coverage that are at least 100 bp long. This definition guarantees high specificity of the extracted LNFRs, as only sequences without any nucleosome are selected. We obtained 47,270 and 79,092 LNFRs for activated and resting T cells, respectively. The numbers for all autosomal chromosomes are shown in Table S2.

The distributions of linker lengths are shown in Figure S5. We can see that the lengths of linkers show a smooth, decreasing distribution without any notable peaks. This fact is important for our work, since it rules the potential risk arising from dinucleosomal fragments out: if the linker DNA between two adjacent nucleosomes had remained undigested by the enzyme, the DNA fragment would have had a total length of 300–350 bp, thus it would have been filtered out and would not have been sequenced [1]. If this had happened frequently, we would have observed a peak in the length distribution of LNFRs around 350–400 bp.

The lengths of LNFRs are similar for resting and activated T cells. However, the LNFRs stemming from resting T cells are slightly longer (mean: 154 bp; median: 134 bp) than for activated T cells (mean: 150 bp; median: 130 bp).

3.1 Estimation of Falsely Detected LNFRs

Since all our further investigations are based on LNFRs, it is important to know the reliability of our LNFR extraction procedure. Since we extend all reads to a length of 150 bp, a false LNFR can only occur if a nucleosome does not produce any mappable read. So the estimation of falsely detected LNFRs comes down to computing the rates to which nucleosomes are not covered by any reads.

In a recent paper, Valouev et al. [11] estimated the average linker length to be 56 bp for human T cells. According to this estimate, in total 13,208,379 nucleosomes fit into the unmasked regions of chromosomes 1–22 of hg18 (the sequenced non-`N` regions of hg18, i.e. where reads can be mapped at all; these regions have a total length of 2,681,303,098 bp). For resting T cells, 137,077,836 reads have been mapped to the unmasked regions of chromosomes 1–22 of hg18, which corresponds to an average of 10.4 reads per nucleosome. Assuming that the numbers of reads are Poisson-distributed, the probability that a nucleosome has no read is 3.1×10^{-5}. This rate would entail an estimated number of 410 false LNFRs for resting T cells (i.e. 0.52% of 79,092). For activated T cells, 126,519,785 reads have been mapped to the unmasked regions of chromosomes 1–22 or hg18, corresponding to an average of 9.6 reads per nucleosome. With the same assumptions as for resting T cells, the probability that a nucleosome has no read is 6.9×10^{-5}, yielding an estimated number of 914 false LNFRs for activated T cells (corresponding to 1.93% of 47,270).

We conclude that the number of falsely detected LNFRs is at most in the range of hundreds and the proportion of them is below 2% for both cell types. This is also in accordance with the findings related to the distribution of linker lengths (see above).

3.2 Validation of LNFRs

We performed the following validations to confirm our LNFRs:

Concordance of LNFRs between activated and resting T cells. Although large-scale chromatin remodeling takes place when T cells are being activated, a considerable proportion of LNFRs should remain stable, since a significant proportion of LNFRs are expected to contain nucleosome-repelling patterns. The LNFRs we determined for resting T cells cover 12,196,145 bp (0.45%) of the total 2,681,303,098 bp of unmasked regions of chromosomes 1–22 in hg18. LNFRs of activated T cells cover 7,080,481 bp (0.26%) of the unmasked regions of chromosomes 1–22 in hg18. The size of overlaps between the two LNFR sets is 922,544 bp, which is 28.7 times as large as one would expect if LNFRs of resting and activated T cells were positioned independently. According to Fisher's exact test, this overlap is significantly larger than under the assumption of independence ($p < 5 \times 10^{-324}$).

Concordance of LNFRs with Valouev et al.'s CD4+ nucleosome data. In their recent paper, Valouev et al. [11] study nucleosome positioning for different human cell types. We used their data obtained from in-vivo CD4+ cells (SRA Accessions SRX046662, SRX046663, SRX046664, and SRX046665) to validate our findings on Schones et al.'s data.

Valouev et al. used the same MNase digestion procedure as Schones et al. [1], but the sequencing of the nucleosome ends was done with a different technology (Applied Biosystems SOLiD 2.0) at a much higher coverage. The CD4+ data set consists of 830,534,836 colorspace reads of length 35. We mapped these reads to hg18 using Bowtie [12] using only the first 28 bases and allowing for up to 3 mismatches. Analogously to the alignment procedure we used for Schones et al.'s data, all reads, even non-unique ones, were taken into account to facilitate reliable LNFRs. Using these settings, 421,056,051 reads could be mapped to hg18. Subsequently, we computed nucleosome occupancy profiles and extracted LNFRs in the same we as for Schones et al.'s data. In total, 51,659 LNFRs were found in the unmasked regions of chromosomes 1–22 of hg18. As can be seen in Figure S6A, the distribution of lengths looks similar, though the LNFRs obtained from Valouev et al.'s data are generally longer (mean length: 284 bp, median length: 186 bp). Interestingly, the GC content histogram in Figure S6B indicates an even larger proportion of GC-rich LNFRs than in the data of Schones et al. [1] (compare with Figure 2 in the main manuscript). This once again confirms our finding that there is a considerable proportion of GC-rich LNFRs.

The LNFRs obtained from the CD4+ data of Valouev et al. cover 14,671,115 bp of the unmasked regions of chromosomes 1–22 of hg18 (corresponding to 0.54% of these regions). The overlap of these in-vivo LNFRs with the LNFRs we obtained for resting T cells is 1,519,499 bp, which is 22.8 times as large as if the in-vivo and resting LNFRs were positioned independently ($p < 5 \times 10^{-324}$ according to Fisher's exact test). The overlap of LNFRs obtained for activated T cells with the in-vivo CD4+ LNFRs obtained for Valouev et al.'s data is 648,395 bp, which is 16.7 times as large as if they were positioned independently ($p < 5 \times 10^{-324}$ according to Fisher's exact test). So we again observe highly significant concordances between the in-vivo LNFRs obtained from Valouev et al.'s data and the LNFRs obtained from the data of Schones et al. [1]. Note that an exact match between any of the LNFR data sets was not to be expected, as all three data sets stem from different cell states.

Validation of LNFRs by ChIP-seq data. In recent years, several ChIP-seq data sets have been released that facilitate further validation of our LNFRs.

Barski et al. [13] have studied a large number of histone variants by ChIP-seq. We have used the H2A.Z data set (obtained from Solexa sequencing of in-vivo CD4+ T cells; SRA Accession SRX000139), since it is the largest one and, therefore, allows for a more reliable analysis than the less common histone variants. Our analysis using the BayesPeak package [14,15] yielded 102,827 peaks, 144 centers of which are in LNFRs of resting T cells. If the positions of H2A.Z peaks and LNFRs were independent, the expected number of peak centers in resting LNFRs would be 468. According to an exact binomial test, this difference is significant ($p = 3.5 \times 10^{-69}$). Moreover, the correlation of H2A.Z read coverage and our nucleosome occupancy profiles is also highly significant ($p < 5 \times 10^{-324}$). So we can conclude, as expected, that there is highly significant correspondence between our LNFRs and H2A.Z occupancy.

Schones et al. [1] also provided H3 ChIP-seq data for resting CD4+ T cells (SRA Accession SRX000167). Using the same analysis pipeline as for the H2A.Z data set, we obtained 310,407 peaks. Again the H3 read coverage and our nucleosome occupancy profiles are highly correlated ($p < 5 \times 10^{-324}$). However, we did not observe any significant difference of H3 occupancy between LNFRs and non-LNFR sequences. This may be caused by the fact that the H3 ChIP-seq peaks are very blurred and, therefore, cover too many LNFRs. The two examples in Figure S1 of [1] seem to support this explanation, although this issue would require a deeper investigation.

Furthermore, we investigated ChIP-seq data for two transcription factors. Using the analysis pipeline mentioned above, we obtained 24,074 peaks indicating CTCF binding sites in primary CD4+ T cells (data set published by Cuddapah et al. [16]; SRA Accession SRX003794). 447 centers of those peaks are inside LNFRs of resting T cells. If CTCF binding sites and LNFRs were positioned independently, the expected number of CTCF peak centers inside LNFRs would be 118. According to an exact binomial test, this difference is significant ($p = 2.3 \times 10^{-119}$). Secondly, we obtained 74,766 peaks indicating Sp1 binding sites in GM12878 cells (lymphoblastoid cells infected with the Epstein-Barr Virus; data provided by the HudsonAlpha Institute for Biotechnology within the frame of the ENCODE project [17]; SRA Accession SRX100408). The centers of 505 of those peaks are in LNFRs or resting T cells, whereas only 365 would be expected if Sp1 peaks and LNFRs were positioned independently. This difference is again significant ($p = 2.6 \times 10^{-12}$ according to an exact binomial test). These two results fit to the fact that transcription factors binding sites are enriched in nucleosome-free regions [16, 18, 19].

We also investigated two Pol II ChIP-seq data sets obtained from resting CD4+ T cells. Processing the Pol II data set provided by Barski et al. [20] (SRA Accession SRX012401) resulted in 69,577 peaks indicating Pol II binding sites. 670 centers of those peaks are in LNFRs of resting T cells. If LNFRs and Pol II peak centers were independent, the expected number o Pol II peak centers in LNFRs would be 316. This difference is significant ($p = 2.3 \times 10^{-67}$ according to an exact binomial test). We further analyzed Schones et al.'s ChIP-seq data set of unphosphorylated Pol II binding sites (SRA Accession SRX000170) [1]. This data set resulted in 33,721 peaks indicating binding sites of unphosphorylated Pol II. The expected number of peak centers in LNFRs of resting T cells would be 153. The number of observed peak centers in LNFRs of resting T cells was 421, which is again significantly more ($p = 4.3 \times 10^{-71}$ according to an exact binomial test). These two results fit to the long-known fact that Pol II prefers nucleosome-free binding sites [21, 22].

Evolutionary conservation of LNFRs. Nucleosome-free regions are enriched at conserved DNA regions both in yeast [23] and human [24]. In order test whether this correspondence is confirmed by our data, we investigated the level of evolutionary conservation of the LNFRs using annotations derived from multiple alignments of 44 vertebrate species and measurements of evolutionary conservation using phastCons [25]. Assemblies of genomes from the following 44 species were included in the multiple alignment supplied to phastCons in order to find conserved elements: *Homo sapiens, Vicugna pacos, Dasypus novemcinctus, Otolemur garnettii, Felis catus, Gallus gallus, Pan troglodytes, Bos taurus, Canis lupus familiaris, Tursiops truncatus, Loxodonta africana, Takifugu rubripes, Gorilla gorilla, Cavia porcellus, Erinaceus europaeus, Equus caballus, Dipodomys ordii, Petromyzon marinus, Anolis carolinensis, Callithrix jacchus, Oryzias latipes, Pteropus vampyrus, Myotis lucifugus, Mus musculus, Microcebus murinus, Monodelphis domestica, Pongo pygmaeus abelii, Ochotona princeps, Ornithorhynchus anatinus, Oryctolagus cuniculus, Rattus norvegicus, Macaca mulatta, Procavia capensis, Sorex araneus, Choloepus hoffmanni, Spermophilus tridecemlineatus, Gasterosteus aculeatus, Tarsier syrichta Echinops telfairi, Tetraodon nigroviridis, Tupaia belangeri, Xenopus tropicalis, Taeniopygia guttata, Danio rerio.* The results are reported in the main manuscript. The computation of p-values is based on an exact binomial test of the number of base pairs lying in conserved elements and in LNFRs, respectively.

Our LNFRs are indeed significantly enriched in conserved elements with $p < 5 \times 10^{-324}$ (exact binomial test) for LNFRs obtained from resting T cells and $p = 3.5 \times 10^{-323}$ for activated T cells.

This concordance again confirms a high quality of the data and our approach to extract LNFRs.

3.3 LNFRs Versus Promoter Regions and GC Content

The main manuscript reports figures about the distribution of the GC content of LNFRs and to which extent they overlap with promoter regions. Figure S7 complements these results by relating the GC content of LNFRs with the proportion to which they overlap with promoter regions. The red graphs in Figure S7 show the proportion of LNFRs overlapping with promoter regions [-10 kbp, +1 kbp] in relation to the GC content (with the histograms of Figure 1 superimposed; Figure S7A: LNFRs of resting T cells, Figure S7B: LNFRs of activated T cells). It becomes evident that GC-rich LNFRs tend to overlap with promoters to a larger extent. However, by far not all LNFRs are overlapping with promoters, not even the GC-rich ones.

We investigated whether LNFRs are enriched in promoter regions. We considered the regions [-1 kbp, +1 kbp] and [-100 bp, +100 bp] centered around the transcription start sites of all coding and non-coding transcripts from the table "UCSC Known Genes" as potential promoter regions. Then we computed the numbers of LNFRs that have an overlap with those promoter regions. We calculated the probability that randomly positioned sequences with the same length distribution as the LNFRs overlap with any of the promoter regions. We used this probability in a one-sided binomial test. Its results along with the numbers of expected and observed overlaps, are shown in Tables S3 and S4 for promoter regions [-1 kbp, +1 kbp] and for promoter regions [-100 bp, +100 bp], respectively. For both promoter sizes and both cell states, overlaps of LNFRs appear significantly more often than randomly. For sequences that were detected as LNFRs in both cell states, the numbers are much smaller, but the difference between observed and expected random overlaps is also significant. Even though the enrichment of LNFRs in promoter regions is highly significant, the majority of LNFR sequences are outside promoter regions.

4 Identification of Remodeled LNFRs

An LNFR of resting T cells is defined to exhibit remodeling if the nucleosome coverage profile for activated T cells is at least 2 somewhere along this LNFR except its first and last 25 bases. The masking of the ends is necessary to avoid detecting minor shifts of nucleosome ends as remodeling. The threshold of 2 has been chosen to avoid that nucleosome indicated by a single erroneous match can lead to false detections of remodeling. Remodeled LNFRs of activated T cells are determined analogously to those of resting T cells by considering the nucleosome coverage profile of resting T cells. In turn, an upper threshold of 1 has been chosen to identify LNFRs that do not show any remodeling.

4.1 Validation of Nucleosome Remodeling by Gene Expression Data

Schones et al. [1] also released gene expression measurements for resting and activated T cells (GEO Accession GSE10437). We used those data to validate the chromatin remodeling sites that we identified as mentioned above. The data set consists of four microarray measurements performed using the Affymetrix Human Genome U133 Plus 2.0 platform, two replicates for each of the two cell states. Raw data were pre-processed using RMA [26]. Since the data set consists of

only four microarrays, it is not meaningful to use methods, such as, LIMMA [27] for determining differentially expressed genes. Instead, we used three simple gene-wise scores to evaluate the difference between expression levels in the two types of T cells: (1) the differences of mean expressions of activated and resting T cells; (2) the standard t-score; (3) a regularized t-score [28]. All three scores are computed such that a positive score means that a gene has a higher expression value in activated than in resting T cells and a negative score, correspondingly, means that the gene is higher expressed in resting T cells.

We distinguish between *remodeled genes*, i.e. genes for which a remodeled LNFR occurs in the [-1 kbp, +1 kbp] region around the transcription start site, and *non-remodeled genes*, i.e. genes for which no such LNFR occurs in the [-1 kbp, +1 kbp] region around the TSS. Among the remodeled genes, we further consider those for which a GC-rich remodeled LNFR occurs in the promoter [-1 kbp, +1 kbp] region around the TSS. For each of the three scores, we performed multiple statistical tests. Table S5 lists the p-values of four of them (one row per test, one column per score):

1. Hypothesis: score for remodeled genes is significantly lower than for non-remodeled genes; this was tested using a Wilcoxon-Mann-Whitney test.
2. Hypothesis: score for remodeled genes with a GC-rich remodeled LNFR in the promoter is significantly lower than for non-remodeled genes; this was tested using a Wilcoxon-Mann-Whitney test too.
3. Hypothesis: remodeled genes are enriched in the set of the 10% most down-regulated genes; this was tested using Fisher's exact test.
4. Hypothesis: remodeled genes with a GC-rich remodeled LNFR in the promoter are enriched in the set of the 10% most down-regulated genes; this was tested using Fisher's exact test too.

As can be seen from Table S5, all four tests indicate significance for all three scores. Not surprisingly, the converse tests for up-regulation of genes were not significant (all p-values were 1). So these tests confirm that the placement of a nucleosome in an otherwise nucleosome-free region of the promoter region of a gene tends to down-regulate this gene.

5 Characterization of Remodeled LNFR Sequences

We used support vector machines in combination with the spectrum kernel in order to extract nucleotide patterns that are specific to DNA loci where chromatin is remodeled via nucleosome repositioning. In this section, we describe the details of our approach.

5.1 Construction of Negative Sets

In order to characterize remodeled LNFRs — considered as *positive samples* in the classification tasks in the following —, it is necessary to construct a set of negative or control sequences, i.e., random sequences that are not remodeled LNFRs. To this end, we drew random sequences from hg18 that do not overlap with LNFRs of the cell state under consideration. In order to ensure

that the classifier can only make use of patterns that are indeed specific to the remodeled LNFRs under consideration, the negative sets have been constructed such that both the sequence length distributions and the GC content distributions are the same as for the respective positive set. This was accomplished by drawing a vast number of random non-LNFR sequences with the same distribution of sequence lengths. From this large pool of negative sequences, a sub-population was drawn such that the same GC content distribution as in the positive set was achieved. The number of negative sequences was always chosen equal to the respective positive set in order to avoid that the classifier is biased towards either class. Finally, we obtained four data sets:

1. remodeled GC-rich LNFRs of resting T cells,
2. remodeled AT-rich LNFRs of resting T cells,
3. remodeled GC-rich LNFRs of activated T cells, and
4. remodeled AT-rich LNFRs of activated T cells.

All four sets, together with the negative sets, were created according to the above procedure. For all four sets of remodeled LNFRs, we trained support vector machines (SVMs) [29, 30] using the spectrum kernel [31].

5.2 Extraction of Indicative DNA Sequence Patterns

A support vector machine (SVM) performs model selection based on a training set (x_i, y_i) $(i = 1, \ldots, l)$, where x_i are the training sequences and y_i are the labels ($y_i = +1$ for a remodeled LNFR / $y_i = -1$ for a random non-LNFR sequence). The selected model is given by the discriminate function

$$g(x) = b + \sum_{i=1}^{l} \alpha_i \cdot y_i \cdot k(x, x_i) \ ,$$

where the offset value b and the non-negative weights α_i are determined via model selection. The two-place function k is called *kernel*; it measures the similarity between two sequences. If $g(x)$ is positive for a new sequence x, the model predicts x as positive, otherwise as negative.

The standard spectrum kernel is defined as

$$k(x, y) = \sum_{p} N(p, x) \cdot N(p, y),$$

where $N(p, x)$ denotes the number of occurrences of pattern p in sequence x [31]. The spectrum kernel for DNA sequences considers all nucleotide sequences of length K, thus the above sum has 4^K summands, so the spectrum kernel computes the similarity of two sequences as the number of sub-sequences of length K they have in common, counting multiple occurrences appropriately. In order to correct for different sequence lengths, we use the *normalized spectrum kernel*

$$k(x, y) = \frac{\sum_p N(p, x) \cdot N(p, y)}{\sqrt{\sum_p N(p, x)^2} \sqrt{\sum_p N(p, y)^2}},$$

which gives a minimum value of 0 if x and y do not share any sub-sequences of length K and a maximum value of 1 if x and y have exactly the same set of sub-sequences of length K.

The (normalized) spectrum kernel facilitates easy extraction of *pattern weights* from an SVM classifier if we make the following rearrangements of the discriminate function [32, 33]:

$$g(x) = b + \sum_{i=1}^{l} \alpha_i \cdot y_i \cdot k(x, x_i) = b + \sum_{i=1}^{l} \alpha_i \cdot y_i \cdot \frac{\sum_p N(p,x) \cdot N(p,x_i)}{\sqrt{\sum_p N(p,x)^2} \sqrt{\sum_p N(p,x_i)^2}}$$

$$= b + \frac{1}{\sqrt{\sum_p N(p,x)^2}} \sum_p N(p,x) \cdot \underbrace{\sum_{i=1}^{l} \alpha_i \cdot y_i \cdot \frac{N(p,x_i)}{\sqrt{\sum_p N(p,x_i)^2}}}_{=w(p)} . \quad (1)$$

So we obtain $w(p)$ as the linear scaling factor with which every occurrence of p in the sequence x is weighted. If $w(p)$ is positive, the pattern p is indicative for the positive class and, if $w(p)$ is negative, p is indicative for the negative class or, equivalently, under-represented in the positive class. The higher the absolute value $w(p)$, the more relevant the pattern p is for the given classification task.

5.3 Computation of Prediction Profiles and Identification of Indicative Sequence Patterns

As described above, the pattern weights $w(p)$ give some insights into which sub-sequences are indicative for the two classes. However, the length of all sub-sequences is fixed to K. That a certain K gives the best cross validation accuracy only means that this K adjusts the model complexity in the best way to balance underfitting and overfitting, but that does not necessarily mean that all patterns indicative for either class are exactly of length K. To elicit patterns indicative for nucleosome remodeling sites independent of the sub-sequence length K, we advocate the following approach: we first compute prediction profiles for all remodeled LNFR sequences and extract sub-sequences, so-called *regions of interest*, that the SVM considers most indicative. Then we apply the MEME motif finder [34, 35] to extract sequence patterns from the hot spots.

Computation of prediction profiles. As can be seen from Eq. (1), the discriminant function of the SVM is essentially a sum of pattern weights, where each sub-sequence p contained in the sequence contributes $w(p)$ to this sum. So we can reformulate the discriminant function $g(x)$ such that each position or base i in the sequence x is attributed the weight s_i it contributes to the discriminant function (i.e. $\frac{1}{K}$ times the sum of the weights of all patterns of which it is part):

$$g(x) = b + \frac{1}{\sqrt{\sum_p N(p,x)^2}} \sum_p N(p,x) \cdot w(p)$$

$$= b + \frac{1}{\sqrt{\sum_p N(p,x)^2}} \sum_{i=1}^{L} \underbrace{\frac{1}{K} \cdot \sum_{j=\max(1,i-K+1)}^{\min(i,L-K+1)} w(x[j,\ldots,j+K-1])}_{=s_i} ,$$

where $x[j,\ldots,j+K-1]$ denotes the sub-sequence of x that starts from the j-th base and ends at base $j-K+1$ (having a total length of K). This approach for computing per-base contributions to the discriminant function is analogous to the one introduced in [33] for the coiled coil kernel.

The region 131,891,956–131,892,186 of chromosome 10 is a GC-rich remodeled LNFR in resting T cells that does not overlap with any promoter region. Figure S8 shows the prediction profiles of the sub-region 131,892,094–131,892,186 for $K = 5,\ldots,9$. It is obvious that, the larger K, the smoother the prediction profile. In any case, the five profiles agree on the fact that the region marked by the gray background is typical for the positive class (remodeled GC-rich LNFRs).

Extraction of regions of interest and identification of typical motifs. We identified sub-sequences most typical for remodeled GC-rich LNFRs as contiguous sub-sequences where the prediction profiles exceed a threshold of 0.2 on a stretch of at least 15 bases. As an example, the region marked in gray in Figure S8 is such a region of interest according to the prediction profile for $K = 9$. Applying this extraction procedure to all remodeled GC-rich LNFRs resulted in 166, 290, 421, 425, and 385 regions of interest for K's of 5, 6, 7, 8, and 9, respectively. For all these five sets of regions of interest, we applied MEME [34,35] to identify common motifs. We used the MEME command line program, version 4.8.1 (released February 7, 2012) to find motifs of lengths 7–20 in the regions of interest or their reverse complements. In order to filter out spurious motifs, we chose an e-value cut-off of 10^{-7} and required each motif to occur at least $0.2 \cdot R$ times, where R is the number of regions of interest. We allowed MEME to consider any number of repetitions of the motif in the sequence, since this gives the most accurate results according to the authors' recommendation. Note that our strategy, although MEME is an unsupervised method, will only produce motifs that are indeed indicative for remodeled GC-rich LNFRs, since our regions of interest are exclusively made up of sub-sequences that are highly typical for remodeled GC-rich LNFRs. The resulting pattern for the regions of interest determined from the profiles obtained for $K = 5$ (the choice of K that gave the best cross validation accuracy) is reported and discussed in the main paper. The resulting patterns for other choices of K are shown in Figure S9.

Validation of motif extraction procedure. In order to make sure that motifs extracted by the above procedure generalize to "future", i.e. previously unseen, sequences, we performed two-fold cross validation. We split our data set (consisting of GC-rich non-promoter remodeled sequences and corresponding negative sequences with equal length and GC content distributions) into two subsets and ran SVM training and motif extraction on each of the two subsets. Table S6 shows the results. In all but one case ($K = 9$ on fold no. 2), only one motif has been extracted from the regions of interest. Of the 11 motifs, 8 are significantly enriched in the positive set both on the training fold they were determined from and the respective test fold. For regions of interest that were determined from the SVMs trained on fold no. 2 for $K = 5,6,7$, in each case, the same motif has been extracted, This motif is not significantly enriched in the positive class, neither on fold no. 2 nor on fold no. 1. So, in the majority of cases, the motif extraction procedure produces significant motifs and, in case they are significantly enriched on the training set, they are also significantly enriched on the test fold. We conclude that the motif extraction procedure produces motifs that generalize well to previously unseen data, which ensures that the patterns are not over-specialized to the data.

6 Nucleosome Remodeling and Methylation

We report in the main manuscript that nucleosome remodeling in human CD4+ T cells is associated with CpG islands. In CpG islands, CpG sites are typically unmethylated, whereas they are typically methylated outside of CpG islands. In order to obtain further confirmation of our findings, we analyzed methylation data. Choi et al. [36] have sequenced methylated DNA fragments of human T cells. We processed the data (SRA Accession SRX012349) using the same pipeline as for ChIP-seq data (see **Materials and Methods** of main manuscript). This resulted in 61,653 significant peaks with an average length of 260 bp and a median length of 151 bp. We considered all `GC` dinucleotides in these peaks as methylated CpG sites, leading to a total number of 1,624,944 such sites.

We compared the ratios to which certain groups of LNFRs contain a methylated CpG sites for different levels of GC content (in order to sort out mere GC content effects, analogously to the results shown in Figure 6 in the main manuscript). Figure S10A shows the results for LNFRs of resting T cells. While the methylation ratio for remodeled LNFRs is not generally lower then for non-remodeled LNFRs (recall that the ratio of remodeled LNFRs that overlap with CpG islands is generally larger than for non-remodeled LNFRs), we can see that, for a GC content around 80%, the ratio of methylated non-remodeled LNFRs is indeed much higher than the proportion of methylated remodeled LNFRs. Note that, for resting T cells, the largest difference in overlaps with CpG islands occurs at a GC content of around 80% (compare with Figure 6). All these statements hold regardless of whether we restrict to non-promoter LNFRs or whether we include promoter LNFRs. In any case, the ratios of methylated LNFRs are higher for non-promoter LNFRs than for promoter LNFRs, which fits well to the fact that more promoter LNFRs overlap with CpG islands than non-promoter LNFRs. Figure S10B shows the results for LNFRs of activated T cells. For these data, no clear difference between any group of LNFRs becomes evident. For a GC content up to 75%, the rates of methylated LNFRs seem to be much lower for activated T cells than for resting T cells. This finding is remarkable in the light of the fact that also the rates of LNFRs overlapping with CpG islands is lower for activated than for resting T cells. The high methylation rates for LNFRs with a GC content around 80% is remarkable as well, but it has to be pointed out that only a small number of 73 LNFRs of activated T cells exceed a GC content of 75%.

To summarize, the results concerning methylation fit well to our findings about the association between nucleosome remodeling and CpG islands, although the differences between remodeled and non-remodeled LNFRs in terms of methylation are not as pronounced as for the overlaps with CpG islands. The reason for this may be twofold: (1) the methylation peaks determined from Choi et al.'s data are rather wide, so we might actually have detected a considerable proportion of false positive methylated CpG sites; (2) there is a considerable proportion of LNFRs that partly overlap with a CpG island while being methylated elsewhere.

7 Homology Analysis of KDM2A and CFP1

In order to study (dis-)similarities between human and mouse versions of KDM2A and CFP1, we downloaded confirmed amino acid sequences of the four proteins from Uniprot [37]:

Q9Y2K7: complete KDM2A sequence, Homo sapiens, 1,162 residues;

P59997: complete KDM2A sequence, Mus musculus, 1,161 residues;

Q9P0U4: complete CFP1 sequence, Homo sapiens, 656 residues;

Q9CWW7: complete CFP1 sequence, Mus musculus, 660 residues;

We performed a pairwise global alignment for both pairs of homologs using the Needleman-Wunsch algorithm [38] as implemented in the EMBOSS suite [39] using the BLOSUM90 scoring matrix [40] and default gap penalties. The choice of the scoring matrix and the penalties is not crucial, since, as we will see next, the sequence similarities are so high that the results are robust against changes of the settings.

Figure S11 shows the results for KDM2A. It is obvious that the two versions are highly similar. As the sequence of human KDM2A is one residue longer, there is one single-residue gap in the alignment. Of the aligned residues, 1,144 are similar (98.5%) and 1,129 are even identical (97.2%). The CXXC domain is free of any mismatches.

Figure S12 shows the results for CFP1, which also appears to occur in very similar forms in humans and mice. The sequence of CFP1 is four residues shorter in humans than in mice, so there is one indel in the alignment which is four residues long. Of the aligned residues, 638 are similar (96.7%) and 635 are even identical (96.2%). Again, the CXXC domain is free of any mismatches.

Supplementary References

1. Schones DE, Cui K, Cuddapah S, Roh TY, Barski A, et al. (2008) Dynamic regulation of nucleosome positioning in the human genome. Cell 132: 887–898.

2. Li R, Li Y, Kristiansen K, Wang J (2008) SOAP: short oligonucleotide alignment program. Bioinformatics 24: 713–714.

3. Dohm JC, Lottaz C, Borodina T, Himmelbauer H (2008) Substantial biases in ultra-short read data sets from high-throughput DNA sequencing. Nucleic Acids Res 36: e105.

4. The 1000 Genomes Project Consortium (2010) A map of human genome variation from population-scale sequencing. Nature 467: 1061–1073.

5. Fan X, Zarmik M, Jin Y, Zhang Y, Liu XS, et al. (2010) Nucleosome depletion at yeast terminators is not intrinsic and can occur by a transcriptional mechanism linked to 3'-end formation. Proc Natl Acad Sci USA 107: 17945–17950.

6. Field Y, Kaplan N, Fondufe-Mittendorf Y, Moore IK, Sharon E, et al. (2008) Distinct modes of regulation by chromatin encoded through nucleosome positioning signals. PLoS Comput Biol 4: e1000216.

7. Mavrich TN, Ioshikhes IP, Venters BJ, Jiang C, Tomsho LP, et al. (2008) A barrier nucleosome model for statistical positioning of nucleosomes throughout the yeast genome. Genome Res 18: 1073–1083.

8. Lee W, Tillo D, Bray N, Morse RH, Davis RW, et al. (2007) A high-resolution atlas of nucleosome occupancy in yeast. Nat Genet 39: 1235–1244.

9. Ioshikhes IP, Albert I, Zanton SJ, Pugh BF (2006) Nucleosome positions predicted through comparative genomics. Nat Genet 38: 1210–1215.

10. Ozsolak F, Song JS, Liu XS, Fisher DE (2007) High-throughput mapping of the chromatin structure of human promoters. Nat Biotechnol 25: 244–248.

11. Valouev A, Johnson SM, Boyd SD, Smith CL, Fire AZ, et al. (2011) Determinants of nucleosome organization in primary human cells. Nature 474: 516–520.

12. Langmead B, Trapnell C, Pop M, Salzberg SL (2009) Ultrafast and memory-efficient alignment of short DNA sequences to the human genome. Genome Biol 10: R25.

13. Barski A, Cuddapah S, Cui K, Roh TY, Schones DE, et al. (2007) High-resolution profiling of histone methylations in the human genome. Cell 129: 823–837.

14. Spyrou C, Stark R, Lynch AG, Tavaré S (2009) BayesPeak: Bayesian analysis of ChIP-seq data. BMC Bioinformatics 10: 299.

15. Cairns J, Spyrou C, Stark R, Smith ML, Lynch AG, et al. (2011) BayesPeak—an R package for analysing ChIP-seq data. Bioinformatics 27: 713–714.

16. Cuddapah S, Johti R, Schones DE, Roh TY, Cui K, et al. (2009) Global analysis of the insulator binding protein CTCF in chromatin barrier regions reveals demarcation of active and repressive domains. Genome Res 19: 24–32.

17. The ENCODE Project Consortium (2004) The ENCODE (ENCyclopedia Of DNA Elements) Project. Science 306: 636–640.

18. Lee C, Shibata Y, Rao B, Strahl B, Lieb J (2004) Evidence for nucleosome depletion at active regulatory regions genome-wide. Nat Genet 36: 900–905.

19. Segal E, Fondufe-Mittendorf Y, Chen L, Thøaström AC, Field Y, et al. (2006) A genomic code for nucleosome positioning. Nature 442: 772–778.

20. Barski A, Jothi R, Cuddapah S, Cui K, Roh TY, et al. (2009) Chromatin poises miRNA- and protein-coding genes for expression. Genome Res 19: 1742–1751.

21. Sakuma K, Matsumura Y, Shensu T (1984) Formation of transcribing mononucleosome-eukaryotic RNA polymerase II complexes in vitro as a simple model of active chromatin. Nucleic Acids Res 12: 1415–1426.

22. Clark DJ (2001) Nucleosome positioning, nucleosome spacing and the nucleosome code. J Biomol Struct Dyn 27: 713–894.

23. Yuan GC, Liu YJ, Dion MF, Slack MD, Wu LF, et al. (2005) Genome-scale identification of nucleosome positions in S. cerevisiae. Science 309: 626–630.

24. Zhang Y, Shin H, Song JS, Lei Y, Liu X (2008) Identifying positioned nucleosomes with epigenetic marks in human from ChIP-Seq. BMC Genomics 9: 537.

25. Siepel A, Bejerano G, Pedersen JS, Hinrichs AS, Hou M, et al. (2005) Evolutionarily conserved elements in vertebrate, insect, worm, and yeast genomes. Genome Res 15: 1034–1050.

26. Irizarry RA, Bolstad BM, Collin F, Cope LM, Hobbs B, et al. (2003) Summaries of Affymetrix GeneChip probe level data. Nucleic Acids Res 31: e15.

27. Smyth GK (2005) Limma: linear models for microarray data. In: Gentleman R, Carey V, Dudoit S, Irizarry R, Huber W, editors, Bioinformatics and Computational Biology Solutions using R and Bioconductor, New York: Springer. pp. 397–420.

28. Baldi P, Long AD (2001) A Bayesian framework for the analysis of microarray expression data: regularized t-test and statistical inferences of gene changes. Bioinformatics 17: 509–519.

29. Cortes C, Vapnik VN (1995) Support vector networks. Machine Learning 20: 273–297.

30. Schölkopf B, Tsuda T, Vert JP (2004) Kernel Methods in Computational Biology. Cambridge, MA: MIT Press.

31. Leslie C, Eskin E, Noble WS (2002) The spectrum kernel: a string kernel for SVM protein classification. In: Altman RB, Dunker AK, Hunter L, Lauderdale K, Klein TED, editors, Pacific Symposium on Biocomputing 2002, World Scientific. pp. 566–575.

32. Bodenhofer U, Schwarzbauer K, Ionescu M, Hochreiter S (2009) Modeling position specificity in sequence kernels by fuzzy equivalence relations. In: Carvalho JP, Dubois D, Kaymak U, Sousa JMC, editors, Proc. Joint 13th IFSA World Congress and 6th EUSFLAT Conference. Lisbon, pp. 1376–1381.

33. Mahrenholz CC, Abfalter IG, Bodenhofer U, Volkmer R, Hochreiter S (2011) Complex networks govern coiled coil oligomerization — predicting and profiling by means of a machine learning approach. Mol Cell Proteomics 10: M110.004994.

34. Bailey TL, Elkan C (1994) Fitting a mixture model by expectation maximization to discover motifs in biopolymers. In: Proc. 2nd Int. Conf. on Intelligent Systems for Molecular Biology. AAAI Press, pp. 28–36.

35. Bailey TL, Bodén M, Buske FA, Frith M, Grant CE, et al. (2009) MEME SUITE: tools for motif discovery and searching. Nucleic Acids Res 37: W202–W208.

36. Choi JK, Bae JB, Lyu J, Kim TY, Kim YJ (2009) Nucleosome deposition and DNA methylation at coding region boundaries. Genome Biol 10: R89.

37. The UniProt Consortium (2012) Reorganizing the protein space at the Universal Protein Resource (UniProt). Nucleic Acids Res 40: D71–D75.

38. Needleman SB, Wunsch CD (1970) A general method applicable to the search for similarities in the amino acid sequence of two proteins. J Mol Biol 48: 443–453.

39. Rice P, Longden I, Bleasby A (2000) EMBOSS: the European Molecular Biology Open Software Suite. Trends Genet 16: 276–277.

40. Henikoff S, Henikoff JG (1992) Amino acid substitution matrices from protein blocks. Proc Natl Acad Sci USA 89: 10915–10919.
