# Supplementary figures and images for: Genome-Wide Chromatin Remodeling Identified at GC-Rich Long Nucleosome-Free Regions
Source: PLoS One. 2012 Nov 5;7(11):e47924. doi: 10.1371/journal.pone.0047924 (PMC3489898; doi:10.1371/journal.pone.0047924)

# GC content of raw sequencing reads

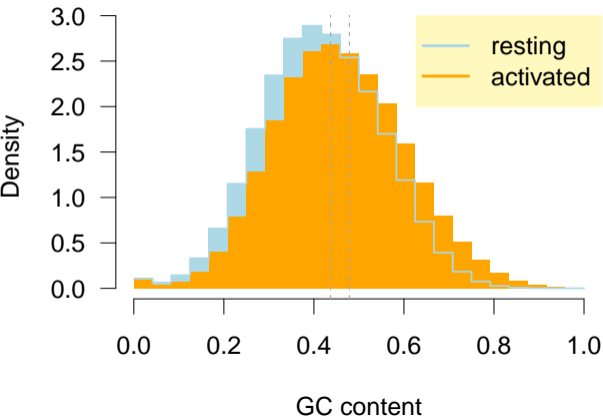

Supplement: Figure S1 — GC content of raw sequencing reads. The average GC content of raw sequence reads is significantly higher for activated (48%) than for resting T cells (44%). (PDF) [file pone.0047924.s002.pdf]

**A**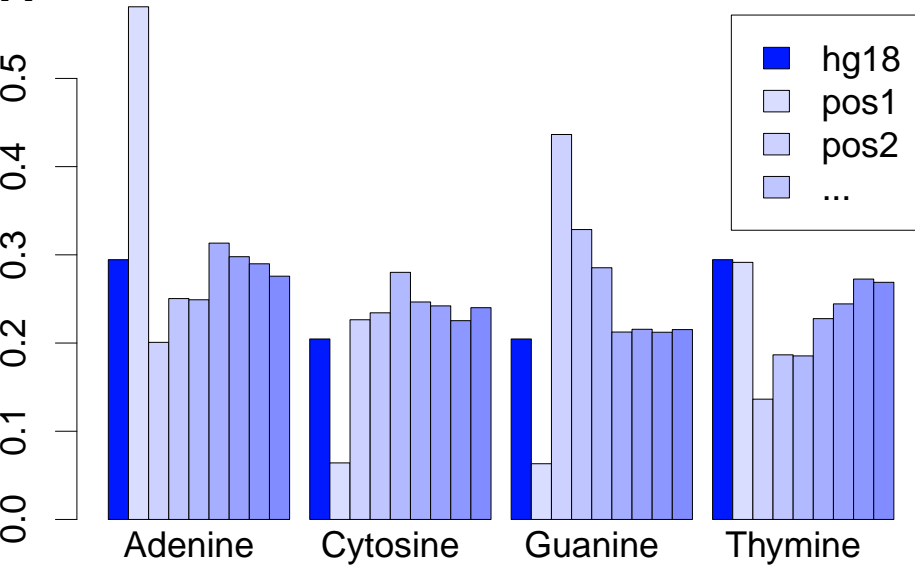**B**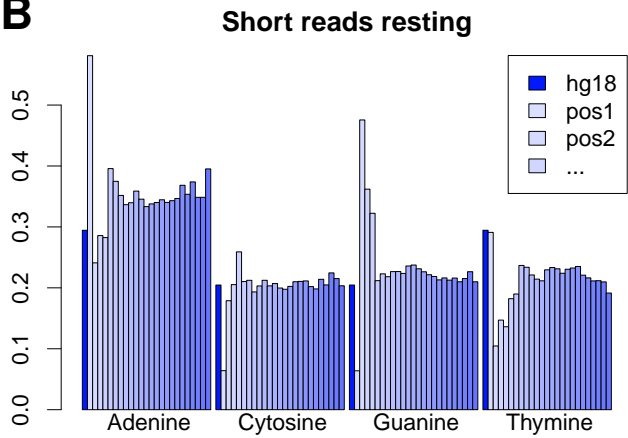**C**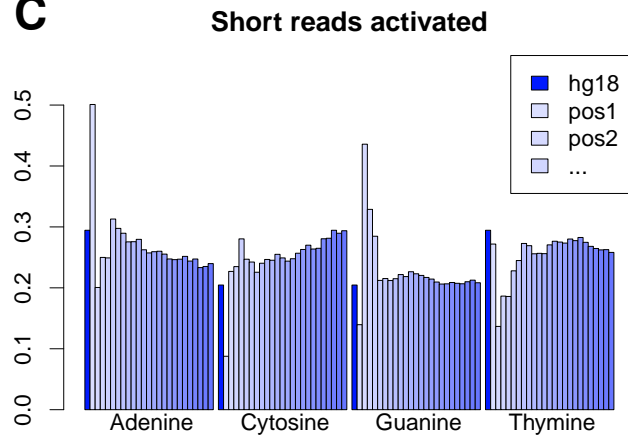

Supplement: Figure S2 — Raw sequence reads are strongly biased to adenine at the first position of the read. Panel A: the average genomic content of nucleotides (dark blue bar labeled “hg18”) is compared to the average content at positions 1–8 of the sequencing reads (light blue bars labeled “pos1”, “pos2”, etc.). Panel B: average genomic nucleotide content versus average nucleotide content at positions 1–24 of reads obtained for resting T cells; Panel B: anologous to panel B for activated T cells. (PDF) [file pone.0047924.s003.pdf]

**A**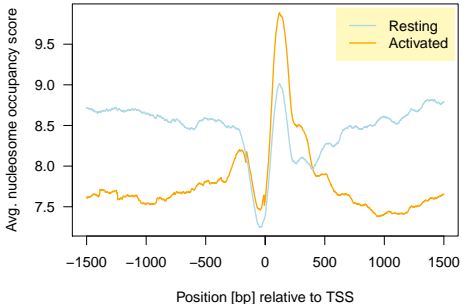**B**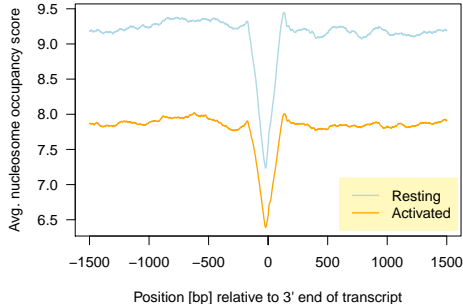

Supplement: Figure S3 — Average nucleosome coverage profiles around the transcription start sites (TSS) and 3′ ends of transcripts. The well-known +1 nucleosome and the 3′ NFR are clearly visible. (PDF) [file pone.0047924.s004.pdf]

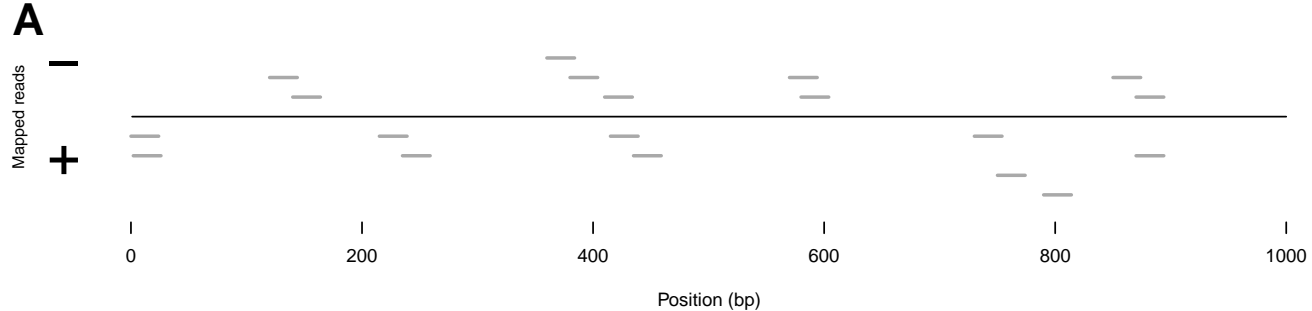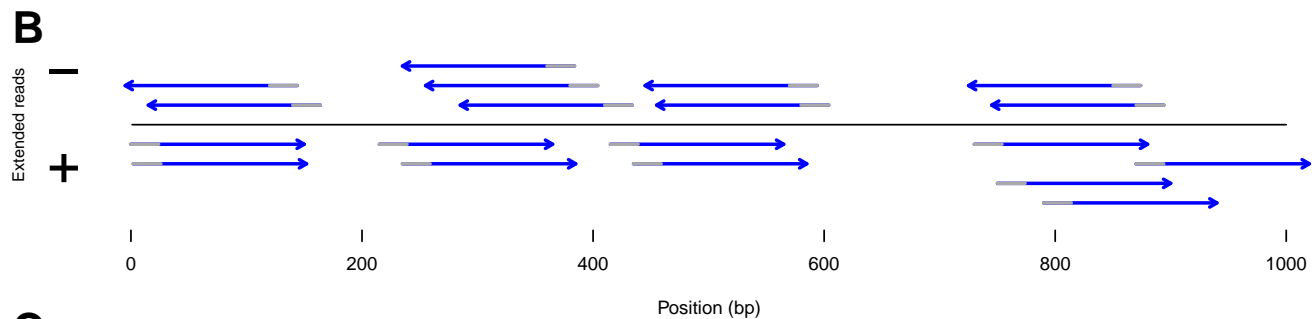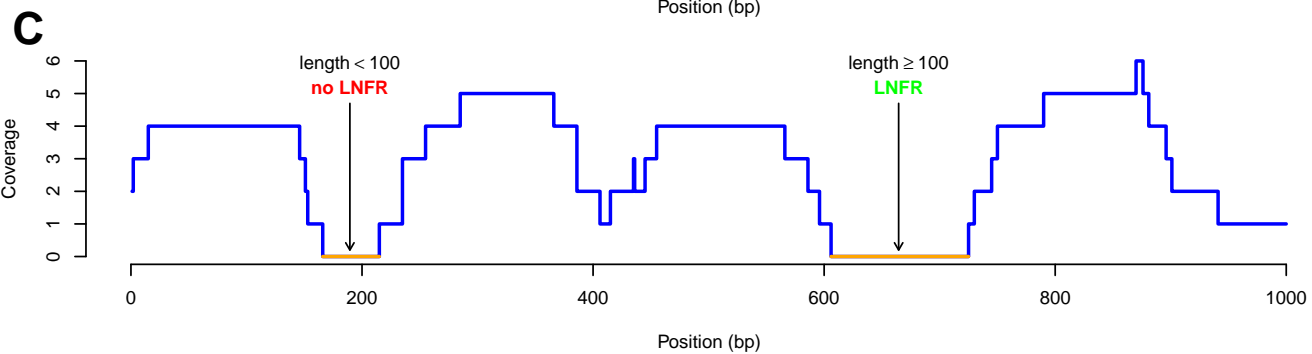

Supplement: Figure S4 — Illustration of computation of nucleosome coverage profiles and LNFR extraction. (PDF) [file pone.0047924.s005.pdf]

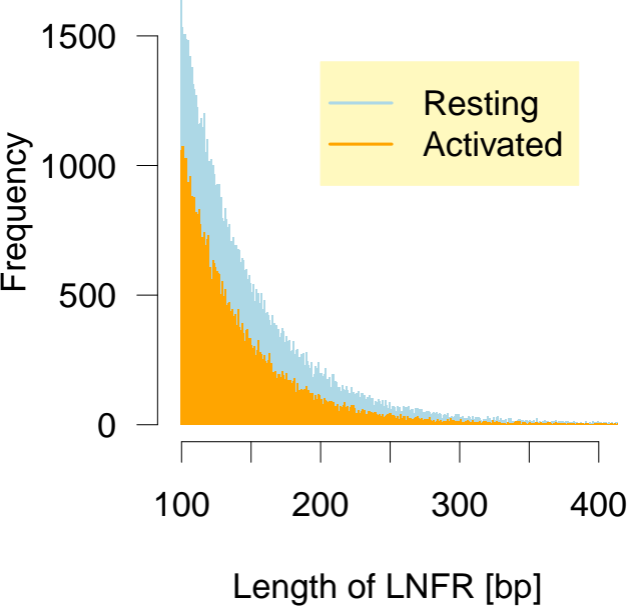

Supplement: Figure S5 — Distributions of LNFR lengths for resting and activated T cells. The length distributions are very similar. Furthermore, no sequencing or biotechnology artifact is visible. (PDF) [file pone.0047924.s006.pdf]

**A**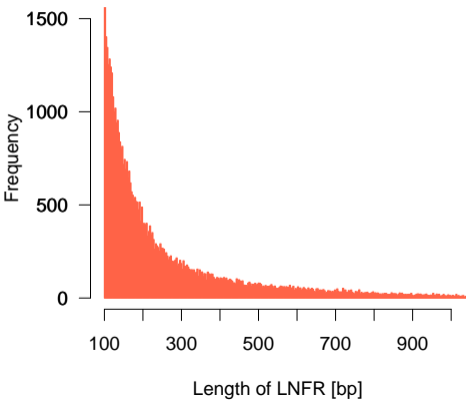**B**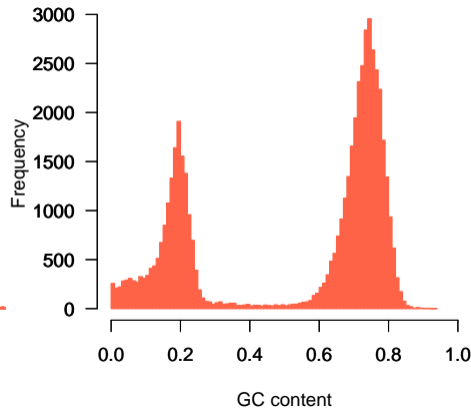

Supplement: Figure S6 — Characteristics of LNFRs extracted from Valouev et al. 's high-coverage data. Panel A shows the distribution of lengths and panel B shows the GC content distribution. (PDF) [file pone.0047924.s007.pdf]

**A**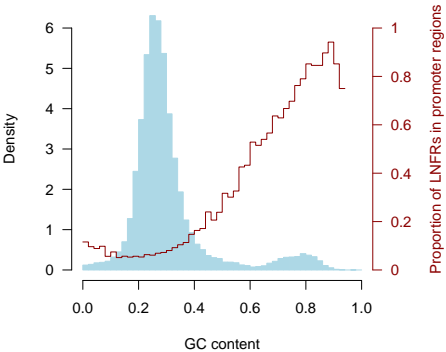**B**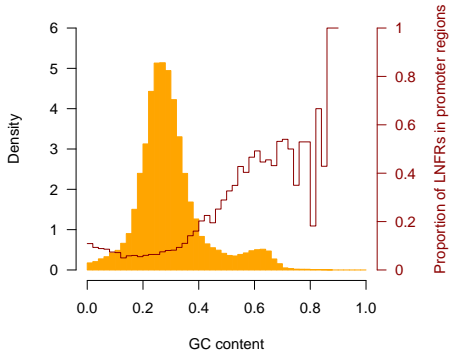

Supplement: Figure S7 — Proportions of LNFRs overlapping with promoter regions [-10 kbp, +1 kbp] (red graphs) versus their GC content. Panel A: data for LNFRs of resting T cells. Panel B: data for LNFRs of activated T cells. The histograms provide the numbers of LNFRs in dependence of GC content. (PDF) [file pone.0047924.s008.pdf]

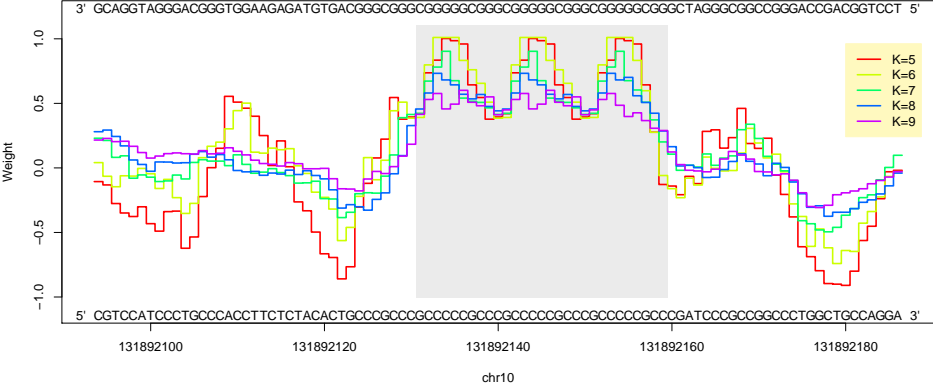

Supplement: Figure S8 — SVM prediction profiles for an exemplary sub-region of an LNFR (pos. 131,892,094–131,892,186 of chromosome 10 in hg18). The larger , the smoother the prediction profile. The five profiles agree on the fact that the region marked by the gray background is typical for the positive class (remodeled GC-rich LNFRs). (PDF) [file pone.0047924.s009.pdf]

**A**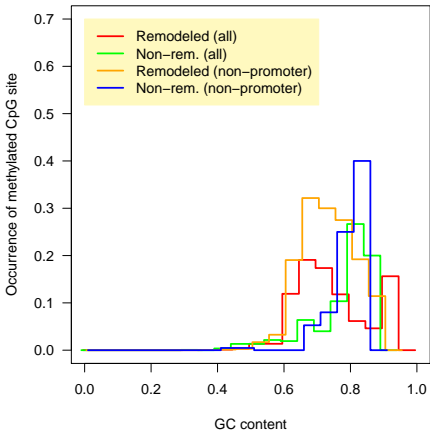**B**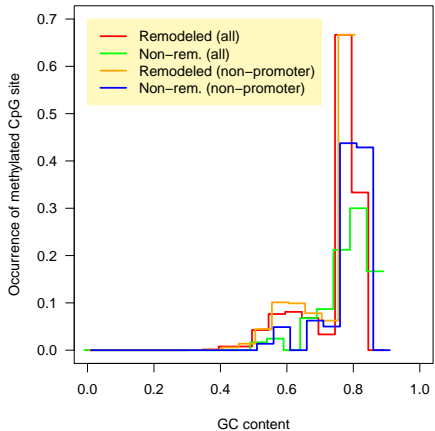

Supplement: Figure S10 — Proportion of LNFRs overlapping with methylation sites plotted versus the LNFRs' GC content. Each curve plots the proportion of LNFRs overlapping with methylation sites in relation to the GC content of the considered LNFRs. For resting T cells (panel A), at a GC content of around 80%, the ratio of methylated non-remodeled LNFRs is indeed much higher than the proportion of methylated remodeled LNFRs. Exactly at this GC content, the largest difference in overlaps with CpG islands occurs (compare with Figure 6). For activated T cells (panel B), the differences are not so evident, but it should be pointed out that, for a GC content up to 75%, the rates of methylated LNFRs are generally lower than for resting T cells. (PDF) [file pone.0047924.s011.pdf]
