# Supplementary material for: Genome-Wide Chromatin Remodeling Identified at GC-Rich Long Nucleosome-Free Regions
Source: PLoS One. 2012 Nov 5;7(11):e47924. doi: 10.1371/journal.pone.0047924 (PMC3489898; doi:10.1371/journal.pone.0047924)
Supplement: Figure S9 — Patterns indicative for remodeled LNFRs obtained from SVM predictions profiles for K's ranging from 6 to 9. The pattern for is shown in Figure 4. (PDF) [file pone.0047924.s010.pdf]

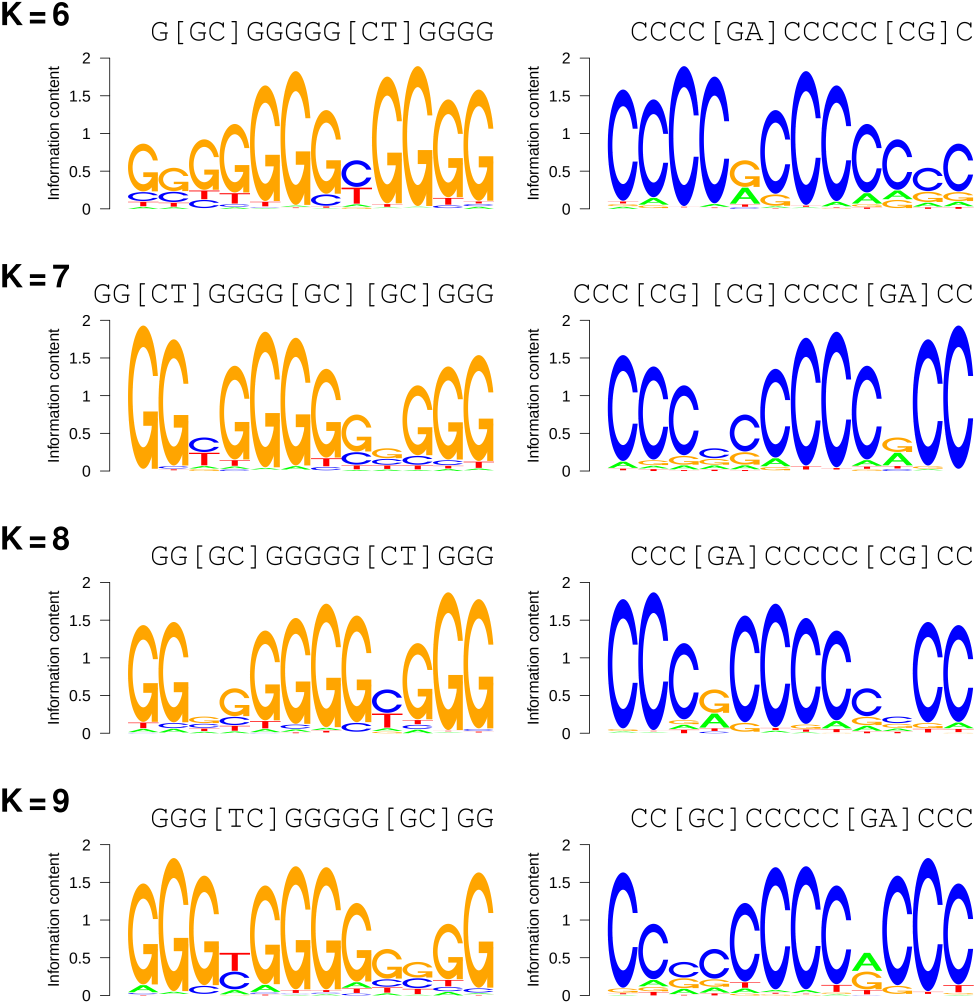
K = 6
G [GC] GGGGG [CT] GGGG
CCCC [GA] CCCCC [CG] C
K = 7
GG [CT] GGGG [GC] [GC] GGG
CCC [CG] [CG] CCCC [GA] CC
K = 8
GG [GC] GGGGG [CT] GGG
CCC [GA] CCCCC [CG] CC
K = 9
GGG [TC] GGGGG [GC] GG
CC [GC] CCCCC [GA] CCC
Information content
0
0.5
1
1.5
2
