# Supplementary material for: Genome-Wide Chromatin Remodeling Identified at GC-Rich Long Nucleosome-Free Regions
Source: PLoS One. 2012 Nov 5;7(11):e47924. doi: 10.1371/journal.pone.0047924 (PMC3489898; doi:10.1371/journal.pone.0047924)
Supplement: Figure S11 — Global alignment of human and mouse KDM2A sequences. There is one single-residue gap. Of the aligned residues, 1,144 are similar (98.5%) and 1,129 are identical (97.2%). The CXXC domain is free of any mismatches. (PDF) [file pone.0047924.s012.pdf]

```
KDM2A (human)   MEPEEERIRYSQRLRGTMRRRYEDDGISDDEIEGKRTFDLEEKLHTNKYN     50
KDM2A (mouse)   MEPEEERIRYSQRLRGTMRRRYEDDGISDDEIEGKRTFDLEEKLQTNKYN     50

KDM2A (human)   ANFVTFMEGKDFNVEYIQRGGLRDPLIFKNSDGLGIKMPDPDFTVNDVKM    100
KDM2A (mouse)   ANFVTFMEGKDFNVEYIQRGGLRDPLIFKNSDGLGIKMPDPDFTVNDVKM    100

KDM2A (human)   CVGSRRMVDVMDVNTQKGIEMTMAQWTRYYETPEEEREKLYNVISLEFSH    150
KDM2A (mouse)   CVGSRRMVDVMDVNTQKGIEMTMAQWTRYYETPEEEREKLYNVISLEFSH    150

KDM2A (human)   TRLENMVQRPSTVDFIDWVDNMWPRHLKESQTESTNAILEMQYPKVQKYC    200
KDM2A (mouse)   TRLENMVQWPSTVDFIDWVDNMWPRHLKESQTESTNAILEMQYPKVQKYC    200

KDM2A (human)   LMSVRGCYTDFHVDFGGTSVWYHIHQGGKVFWLIPPTAHNLELYENWLLS    250
KDM2A (mouse)   LISVRGCYTDFHVDFGGTSVWYHIHQGGKVFWLIPPTAHNLELYENWLLS    250

KDM2A (human)   GKQGDIFLGDRVSDCQRIELKQGYTFVIPSGWIHAVYTPTDTLVFGGNFL    300
KDM2A (mouse)   GKQGDIFLGDRVSDCQRIELKQGYTFVIPSGWIHAVYTPTDTLVFGGNFL    300

KDM2A (human)   HSFNIPMQLKIYNIEDRTRVPNKFRYPFYYEMCWYVLERYVYCITNRSHL    350
KDM2A (mouse)   HSFNIPMQLKIYSIEDRTRVPNKFRYPFYYEMCWYVLERYVYCITNRSHL    350

KDM2A (human)   TKEFQKESLSMDLELNGLESGNGDEEAVDREPRRLSSRRSVLTSPVANGV    400
KDM2A (mouse)   TKDFQKESLSMDMELNELESGNGDEEGVDREARRMNNKRSVLTSPVANGV    400

KDM2A (human)   NLDYDGLGKTCRSLPSLKKTLAGDSSSDCSRGSHNGQVWDPQCAPRKDRQ    450
KDM2A (mouse)   NLDYDGLGKACRSLPSLKKTLSGDSSSDSTRGSHNGQVWDPQCSPKKDRQ    450

KDM2A (human)   VHLTHFELEGLRCLVDKLESLPLHKKCVPTGIEDEDALIADVKILLEELA    500
KDM2A (mouse)   VHLTHFELEGLRCLVDKLESLPLHKKCVPTGIEDEDALIADVKILLEELA    500

KDM2A (human)   NSDPKLALTGVPIVQWPKRDKLKFPTRPKVRVPTIPITKPHTMKPAPRLT    550
KDM2A (mouse)   SSDPKLALTGVPIVQWPKRDKLKFPTRPKVRVPTIPITKPHTMKPAPRLT    550

                                              CXXC
KDM2A (human)   PVRPAAASPIVSGARRRRVRCRKCKACVQGECGVCHYCRDMKKFGGPGRM    600
KDM2A (mouse)   PVRPAAASPIVSGARRRRVRCRKCKACVQGECGVCHYCRDMKKFGGPGRM    600

KDM2A (human)   KQSCVLRQCLAPRLPHSVTCSLCGEVDQNEETQDFEKKLMECCICNEIVH    650
KDM2A (mouse)   KQSCVLRQCLAPRLPHSVTCSLCGEVDQNEETQDFEKKLMECCICNEIVH    650

KDM2A (human)   PGCLQMDGEGLLNEELPNCWECPKCYQEDSSEKAQKRKMEESDEEAVQAK    700
KDM2A (mouse)   PGCLQMDGEGLLNEELPNCWECPKCYQEDSSDKAQKRKIEESDEEAVQAK    700

KDM2A (human)   VLRPLRSCDEPLTPPPHSPTSMLQLIHDPVSPRGMVTRSSPGAGPSDHHS    750
KDM2A (mouse)   VLRPLRSCEEPLTPPPHSPTSMLQLIHDPVSPRGMVTRSSPGAGPSDHHS    750

KDM2A (human)   ASRDERFKRRQLLRLQATERTMVREKENNPSGKKELSEVEKAKIRGSYLT    800
KDM2A (mouse)   ASRDERFKRRQLLRLQATERTMVREKENNPSGKKELSEVEKAKIRGSYLT    800

KDM2A (human)   VTLQRPTKELHGTSIVPKLQAITASSANLRHSPRVLVQHCPARTPQRGDE    850
KDM2A (mouse)   VTLQRPTKELHGTSIVPKLQAITASSANLRPNPRVLMQHCPARNPQHGDE    850

KDM2A (human)   EGLGGEEEEEEEEEEEEDDSAEEGGAARLNGRGSWAQDGDESWMQREVWMS    900
KDM2A (mouse)   EGLGG.EEEEEEEEEEEDDSAEEGGAARLNGRGSWAQDGDESWMQREVWMS    899

KDM2A (human)   VFRYLSRRELCECMRVCKTWYKWCCDKRLWTKIDLSRCKAIVPQALSGII    950
KDM2A (mouse)   VFRYLSRKELCECMRVCKTWYKWCCDKRLWTKIDLSRCKAIVPQALSGII    949

KDM2A (human)   KRQPVSLDLSWTNISKKQLTWLVNRLPGLKDLLLAGCSWSAVSALSTSSC   1000
KDM2A (mouse)   KRQPVSLDLSWTNISKKQLTWLVNRLPGLKDLLLAGCSWSAVSALSTSSC    999

KDM2A (human)   PLLRTLDLRWAVGIKDPQIRDLLTPPADKPGQDNRSKLRNMTDFRLAGLD   1050
KDM2A (mouse)   PLLRTLDLRWAVGIKDPQIRDLLTPPTDKPGQDNRSKLRNMTDFRLAGLD   1049

KDM2A (human)   ITDATLRLIIRHMPLLSRLDLSHCSHLTDQSSNLLTAVGSSTRYSLTELN   1100
KDM2A (mouse)   ITDATLRLIIRHMPLLSRLDLSHCSHLTDQSSNLLTAVGSSTRYSLTELN   1099

KDM2A (human)   MAGCNKLTDQTLIYLRRIANVTLIDLRGCKQITRKACEHFISDLSINSLY   1150
KDM2A (mouse)   MAGCNKLTDQTLFFLRRIANVTLIDLRGCKQITRKACEHFISDLSINSLY   1149

KDM2A (human)   CLSDEKLIQKIS   1162
KDM2A (mouse)   CLSDEKLIQKIS   1161
```
