# Supplementary material for: Genome-Wide Chromatin Remodeling Identified at GC-Rich Long Nucleosome-Free Regions
Source: PLoS One. 2012 Nov 5;7(11):e47924. doi: 10.1371/journal.pone.0047924 (PMC3489898; doi:10.1371/journal.pone.0047924)
Supplement: Figure S12 — Global alignment of human and mouse CFP1 sequences. There is one four-residue indel. Of the aligned residues, 638 are similar (96.7%) and 635 are even identical (96.2%). The CXXC domain is free of any mismatches. (PDF) [file pone.0047924.s013.pdf]

```
CFP1 (human)   MEGDGSDPEPPDAGEDSKSENGENAPIYCICRKPDINCFMIGCDNCNEWF   50
CFP1 (mouse)   MEGDGSDLEPPDAGDDSKSENGENAPIYCICRKPDINCFMIGCDNCNEWF   50

CFP1 (human)   HGDCIRITEKMAKAIREWYCRECREKDPKLEIRYRHKKSRERDGNERDSS  100
CFP1 (mouse)   HGDCIRITEKMAKAIREWYCRECREKDPKLEIRYRHKKCRERDGSERAGS  100

CFP1 (human)   EPRDEGGGRKRPVPDPDLQRRAGSGTGVGAMLARGSASPHKSSPQPLVAT  150
CFP1 (mouse)   EPRDEGGGRKRPASDPELQRRAGSGTGVGAMLARGSASPHKSSPQPLVAT  150
                                                 CXXC
CFP1 (human)   PSQHH....QQQQQQIKRSARMCGECEACRRTEDCGHCDFCRDMKKFGGP  196
CFP1 (mouse)   PSQHHHQQQQQQQQQIKRSARMCGECEACRRTEDCGHCDFCRDMKKFGGP  200

CFP1 (human)   NKIRQKCRLRQCQLRARESYKYFPSSLSPVTPSESLPRPRRPLPTQQQPQ  246
CFP1 (mouse)   NKIRQKCRLRQCQLRARESYKYFPSSLSPVTPSEALPRPRRPPPTQQQPQ  250

CFP1 (human)   PSQKLGRIREDEGAVASSTVKEPPEATATPEPLSDEDLPLDPDLYQDFCA  296
CFP1 (mouse)   QSQKLGRIREDEGTVLSSVVKEPPEATATPEPLSDEDLALDPDLYQDFCA  300

CFP1 (human)   GAFDDHGLPWMSDTEESPFLDPALRKRAVKVKHVKRREKKSEKKKEERYK  346
CFP1 (mouse)   GAFDDHGLPWMSDAEESPFLDPALRKRAVKVKHVKRREKKSEKKKEERYK  350

CFP1 (human)   RHRQKQKHKDKWKHPERADAKDPASLPQCLGPGCVRPAQPSSKYCSDDCG  396
CFP1 (mouse)   RHRQKQKHKDKWKHPERADAKDPASLPQCLGPGCVRAAQPGSKYCSDDCG  400

CFP1 (human)   MKLAANRIYEILPQRIQQWQQSPCIAEEHGKKLLERIRREQQSARTRLQE  446
CFP1 (mouse)   MKLAANRIYEILPQRIQQWQQSPCIAEEHGKKLLERIRREQQSARTRLQE  450

CFP1 (human)   MERRFHELEAIILRAKQQAVREDEESNEGDSDDTDLQIFCVSCGHPINPR  496
CFP1 (mouse)   MERRFHELEAIILRAKQQAVREDEENNENDSDDTDLQIFCVSCGHPINPR  500

CFP1 (human)   VALRHMERCYAKYESQTSFGSMYPTRIEGATRLFCDVYNPQSKTYCKRLQ  546
CFP1 (mouse)   VALRHMERCYAKYESQTSFGSMYPTRIEGATRLFCDVYNPQSKTYCKRLQ  550

CFP1 (human)   VLCPEHSRDPKVPADEVCGCPLVRDVFELTGDFCRLPKRQCNRHYCWEKL  596
CFP1 (mouse)   VLCPEHSRDPKVPADEVCGCPLVRDVFELTGDFCRLPKRQCNRHYCWEKL  600

CFP1 (human)   RRAEVDLERVRVWYKLDELFEQERNVRTAMTNRAGLLALMLHQTIQHDPL  646
CFP1 (mouse)   RRAEVDLERVRVWYKLDELFEQERNVRTAMTNRAGLLALMLHQTIQHDPL  650

CFP1 (human)   TTDLRSSADR   656
CFP1 (mouse)   TTDLRSSADR   660
```
