# Supplementary material for: Genome-Wide Chromatin Remodeling Identified at GC-Rich Long Nucleosome-Free Regions
Source: PLoS One. 2012 Nov 5;7(11):e47924. doi: 10.1371/journal.pone.0047924 (PMC3489898; doi:10.1371/journal.pone.0047924)
Supplement: Table S1 — Statistics of mapping short reads using SOAP for resting and activated T cells. (PDF) [file pone.0047924.s014.pdf]

	resting	activated
number of distinct reads	254,003,438	248,219,348
number of reads mapped	137,077,836	126,519,785
number of reads mapped without mismatch	85,573,678	80,768,331
number of reads mapped with gaps	5,657,788	2,454,046
number of reads mapped with mismatch	45,844,905	43,295,959
number of reads mapped uniquely	115,255,277	107,466,802
number of matches	784,122,919	783,374,099
