# Supplementary material for: Genome-Wide Chromatin Remodeling Identified at GC-Rich Long Nucleosome-Free Regions
Source: PLoS One. 2012 Nov 5;7(11):e47924. doi: 10.1371/journal.pone.0047924 (PMC3489898; doi:10.1371/journal.pone.0047924)
Supplement: Table S2 — Numbers of extracted LNFRs in all autosomal chromosomes in the human genome. (PDF) [file pone.0047924.s015.pdf]

Chromosome	number of LNFRs in resting T cells	number of LNFRs in activated T cells	number of LNFRs in both cell states
chr1	5,714	3,526	283
chr2	7,196	4,397	419
chr3	6,050	3,600	342
chr4	7,880	4,315	485
chr5	6,037	3,412	349
chr6	5,668	3,202	337
chr7	4,592	2,756	295
chr8	4,536	2,679	297
chr9	3,000	1,755	169
chr10	3,190	2,011	191
chr11	3,263	1,960	179
chr12	3,706	2,295	223
chr13	4,049	2,272	205
chr14	2,943	1,849	304
chr15	1,653	1,108	114
chr16	1,530	931	85
chr17	1,415	1,031	75
chr18	2,584	1,485	145
chr19	1,161	738	88
chr20	1,197	822	68
chr21	1,191	722	70
chr22	537	404	38
**total**	**79,092**	**47,270**	**4,761**
