# Supplementary material for: Genome-Wide Chromatin Remodeling Identified at GC-Rich Long Nucleosome-Free Regions
Source: PLoS One. 2012 Nov 5;7(11):e47924. doi: 10.1371/journal.pone.0047924 (PMC3489898; doi:10.1371/journal.pone.0047924)
Supplement: Table S3 — Numbers of LNFRs overlapping with promoter regions [−1 kbp, +1 kbp]. (PDF) [file pone.0047924.s016.pdf]

	number of all LNFRs	observed overlapping LNFRs	expected overlapping LNFRs	*p*-value
Resting	79,092	5,018	2,179	$<5\times10^{-324}$
Activated	47,270	2,002	1,300	2.9×10^{-75}
Resting and activated	4,761	159	132	0.01
