# Supplementary material for: Genome-Wide Chromatin Remodeling Identified at GC-Rich Long Nucleosome-Free Regions
Source: PLoS One. 2012 Nov 5;7(11):e47924. doi: 10.1371/journal.pone.0047924 (PMC3489898; doi:10.1371/journal.pone.0047924)
Supplement: Table S5 — p -values of tests for differential expression of remodeled genes versus non-remodeled genes. All four tests indicate significance for all three scores (details to be found in Text S1, Section 4.1). (PDF) [file pone.0047924.s018.pdf]

**Hypothesis/test**	**diff. of mean expression**	***t*-score**	**regularized *t*-score**
remodeled $<$ non-remodeled (Wilcoxon-Mann-Withney test)	1.7×10^{-17}	2.0×10^{-16}	3.5×10^{-17}
remodeled (GC) $<$ non-remodeled (Wilcoxon-Mann-Withney test)	5.9×10^{-20}	2.5×10^{-18}	1.5×10^{-20}
remodeled enriched in downreg. (Fisher's exact test)	4.9×10^{-14}	8.3×10^{-8}	8.5×10^{-13}
remodeled (GC) enriched in downreg. (Fisher's exact test)	5.1×10^{-13}	2.4×10^{-7}	3.7×10^{-13}
