# Supplementary material for: Genome-Wide Chromatin Remodeling Identified at GC-Rich Long Nucleosome-Free Regions
Source: PLoS One. 2012 Nov 5;7(11):e47924. doi: 10.1371/journal.pone.0047924 (PMC3489898; doi:10.1371/journal.pone.0047924)
Supplement: Table S6 — Results of cross validation analysis of pattern extraction procedure. In the majority of cases, the motif extraction procedure produces significant motifs and, in case they are significantly enriched on the training set, they are also significantly enriched on the test fold. (PDF) [file pone.0047924.s019.pdf]

Training set	K	Motif (reg. exp.)	e-value (MEME)	Test set Fold 1 pos. (%)	Fold 1 neg. (%)	Fold 1 p-value (Fisher test)	Fold 2 pos. (%)	Fold 2 neg. (%)	Fold 2 p-value (Fisher test)
Fold 1	5	G[CG]GGGGG[TC]GGGG	1.2×10^{-53}	7.10	2.48	1.1×10^{-4}	6.45	3.14	5.0×10^{-3}
	6	GGGG[TC]GGGG	7.9×10^{-72}	36.5	16.2	4.7×10^{-16}	36.9	17.2	6.6×10^{-15}
	7	GG[CTA]GGGGG[CGT]GGG	9.2×10^{-105}	10.4	4.95	2.5×10^{-4}	10.1	4.13	3.7×10^{-5}
	8	G[CG]GGGGG[TC]GGGG	3.3×10^{-132}	7.10	2.48	1.1×10^{-4}	6.45	3.14	5.0×10^{-3}
	9	GG[CTA]GGGGG[GCT]GGG	4.2×10^{-134}	10.4	4.95	2.5×10^{-4}	10.1	4.13	3.7×10^{-5}
Fold 2	5	GGGG[CT]GGGGGGG	6.6×10^{-96}	1.32	0.99	0.39	2.14	1.32	0.19
	6	GGGG[TC]GGGGGGG	2.0×10^{-131}	1.32	0.99	0.39	2.14	1.32	0.19
	7	GGGG[TC]GGGGGGG	2.0×10^{-134}	1.32	0.99	0.39	2.14	1.32	0.19
	8	GGG[TCA]GGGGG[GC]GG	1.0×10^{-128}	6.27	3.14	7.0×10^{-3}	8.26	3.47	2.6×10^{-4}
	9	GGGG[CT]GGGG	7.1×10^{-116}	36.5	16.2	4.7×10^{-16}	36.9	17.2	6.6×10^{-15}
		[GA][CG]GGGGG[GC][AC]G[GT]C	1.2×10^{-13}	2.81	0.82	8.0×10^{-3}	3.47	0.99	2.7×10^{-3}
